# Green Synthesis of AgNP-Decorated MWCNTs via *Origanum dubium* for Enhanced Antimicrobial and Antioxidant Activity

**DOI:** 10.3390/molecules31183322

**Published:** 2026-09-19

**Authors:** Erhan Ertekin, Nur Pasaoglulari Aydinlik, Doga Kavaz, Oluwasuyi Ayobami Oba

**Affiliations:** 1 Faculty of Arts and Sciences, Department Basic Sciences and Humanities, Cyprus International University, 99258 Nicosia, Türkiye; eertekin@u.rochester.edu (E.E.); nurp@ciu.edu.tr (N.P.A.); ooba@ciu.edu.tr (O.A.O.); 2 Faculty of Engineering, Department of Bioengineering, Cyprus International University, 99258 Nicosia, Türkiye; 3 Biotechnology Research Center, Cyprus International University, 99258 Nicosia, Türkiye

**Keywords:** green synthesis, functionalized multi-walled carbon nanotube, multi-walled carbon nanotube, silver nanoparticles, bioactivity

## Abstract

A sustainable method has been developed to produce multi-walled carbon nanotubes (MWCNTs) that are adorned with a unique silver nanoparticle, using a plant extract (*Origanum dubium*) as a mediator. Gas chromatography/mass spectrometry (GC/MS) technique was used to investigate the chemical components of the plant extract. Silver nanoparticles (AgNPs) have been deposited on the MWCNTs as well as acid-functionalized MWCNTs (f-MWCNTs) via an in situ solution method. The present study presents a sustainable and economical approach to the synthesis of AgNPs. The biosynthesis of Ag nanoparticles has been optimized by varying several parameters, including pH, temperature, reaction time, concentration of plant extract, and duration of UV exposure. The nanohybrids composed of AgNPs, MWCNTs, acid-treated MWCNTs, and AgNP-decorated MWCNT nanocomposites were successfully synthesized and evaluated for their antibacterial potential against the Gram-positive methicillin-resistant *Staphylococcus aureus* (MRSA) and the Gram-negative *Escherichia coli* (*E. coli*) strains. Their radical scavenging activities were also evaluated against DPPH and hydrogen peroxide. X-ray diffraction (XRD) analysis, Fourier transform infrared spectroscopy (FTIR), and UV–vis spectroscopy were used to investigate the structure and nature of f-MWCNT and decorated nanocomposites. The scanning electron microscopy (SEM) technique was used to analyze the dispersion state of carbon nanotubes and the immobilization of AgNPs. The study reveals that the modified AgNPs possess viable antimicrobial and antioxidant activities.

## 1. Introduction

The construction of hybrid materials combining nanotubes and nanoparticles has garnered increasing attention due to their unusual structure and capabilities, which show promise in the fields of electrocatalysis, biosensors, heterogenous catalysis, biological activities, and nanoelectronics [1,2,3,4,5]. Metal nanoparticle deposition is facilitated by the presence of an outer surface on multi-walled carbon nanotubes (MWCNTs). Nonetheless, strong van der Waals interactions frequently result in the aggregation of carbon nanotubes (CNTs) in aqueous conditions. This results in the formation of large bundles, making CNTs insoluble and restricting their potential applications [6,7,8]. One commonly used method to improve dispersion and maximize the effectiveness of CNTs is through chemical functionalization [9,10,11]. This involves treating the CNTs with acids to introduce hydroxyl and carboxyl functional groups. These nanoparticles have been chemically modified and made soluble using covalent and non-covalent techniques [12,13,14]. The covalent attachment of small or large molecules to CNTs enhances their stability and effectiveness. Grafted molecules increase the solubility of CNTs, even with a low degree of functionalization [15,16,17]. In order to attach carboxylic acid (-COOH) residues on CNTs, reactions with nitric and sulfuric acid are commonly used. These reactions typically involve oxidation at the more reactive or open end or defect sites of the CNTs, rather than their side walls [18,19]. This approach is known for its simplicity and absence of multiple organic synthesis steps. Precursor functionalization commonly involves using the hydroxyl and carboxyl groups found at the ends and defect sites of CNTs to synthesize amine, ester, and organometallic structures [20,21].

Extensive research has focused on attaching metal particles to the structure of CNTs in the field of nanomedicine, specifically for their biomedical uses [2,3]. The decoration of CNTs with noble metal nanoparticles is a fascinating area of research. The electrocatalytic activity of AgNPs on inert and conducting materials has been demonstrated for the oxidation of organic molecules [22]. Due to their broad spectrum of antimicrobial action against bacteria, viruses, and fungi, AgNPs are widely recognized for their potent antibacterial qualities [23,24,25]. However, two critical limitations exist regarding the application of AgNPs in nanomedicine. The first pertains to cytotoxicity, which may lead to chronic toxicity due to the accumulation of AgNPs in essential organs. The second limitation involves the aggregation of AgNPs, which alters their physicochemical properties, ultimately diminishing their bioavailability and therapeutic efficacy [21]. The antibacterial activity of materials can be decreased, and particle aggregation can occur due to the instability of AgNPs in aqueous solution. Furthermore, AgNPs have the potential to be extremely hazardous to human cells and the environment at large doses [10,11,12]. The release of silver ions is responsible for the production of reactive oxygen species (ROS), which is why AgNPs are hazardous [25]. Novel nanostructures have been proposed as alternatives to address the limitations mentioned. One approach is to decorate AgNPs on supporting nanomaterials like CNTs [26].

This approach shows promise for various technological applications. Hybrid materials including CNTs and AgNPs may show a number of appealing characteristics that have not yet been thoroughly investigated [27]. CNTs—more specifically, single-walled and multi-walled CNTs—have gained widespread recognition as useful supporting materials because of their remarkable qualities, which include chemical stability and a high surface area to volume ratio [27]. Due to their vast surface area, CNTs may be used as efficient templates to deposit AgNPs onto MWCNTs. Antimicrobial activity is significantly increased, and stability is improved by this procedure [21]. Prior studies have shown that AgNP-decorated MWCNTs have demonstrated improved antimicrobial properties and reduced cytotoxicity compared to AgNPs alone [28,29,30]. The strong adhesion of nanoparticles on functionalized MWCNTs reduces the toxicity of the AgNPs by preventing their easy release to the cells [28]. This study involves introducing carboxyl and hydroxyl functional groups onto MWCNTs through reflux with nitric and sulfuric acid. The carboxyl and hydroxyl groups on the surface of the MWCNTs were used as specific nucleation sites to chelate and reduce silver ions in situ. This prevented the metal nanoparticles from agglomerating in solution. Various techniques for preparing Ag/MWCNTs include vapor deposition [31,32], surface chemical reduction [33,34,35], and gamma-irradiation [36,37,38,39]. However, none of these methods yield satisfactory results, as, in most cases, the agglomeration of AgNPs is still an issue, and consequently, a reduced number of nanoparticles can be deposited onto the surface of the MWCNTs. In the present study, a plant extract-mediated green synthesis approach was employed for the in situ formation and decoration of AgNPs onto pristine and acid-functionalized MWCNT surfaces.

The genus Origanum, belonging to the family Lamiaceae, encompasses annual, perennial, and shrubby herbaceous plants. It comprises about 15 to 20 species and is widely distributed throughout the world. The essential oils derived from the genus Origanum encompass a variety of active constituents [25]. Their range of antibacterial, antitumor, and immunomodulatory effects is extensive; however, there exists a paucity of research concerning the nonvolatile constituents, such as flavonoids, organic acids, terpenoids, and sterols. The species *Origanum dubium* (Cypriot oregano) was selected due to its recognized potential as a significant source of bioactive compounds. These compounds are progressively utilized in nanotechnology to improve stability, solubility, and efficacy in applications related to antimicrobial activity, antioxidant properties, and food preservation attributed to their high phenolic content [26].

Therefore, this study utilized the plant extracts as the reducing agent to eliminate the use of chemical reducing agents such as dimethylformamide (DMF) and sodium hydroxide (NaOH). In the traditional chemical production of AgNPs, external reducing and/or stabilizing agents, such as sodium borohydride (NaBH_4_), ascorbic acid or ascorbate, dimethylformamide (DMF), and Tollens-type silver–ammonia chemistry, are often added. While these methods can offer efficient control over nucleation and particle growth, this comes at the cost of additional chemical reagents and residues that may require subsequent removal or treatment. NaOH is generally used as a base for pH adjustment rather than as the main reducing agent and was used in the present study only for controlling the pH reaction. Conversely, plant-mediated synthesis uses naturally present phytochemicals, like phenolic compounds, flavonoids, terpenoids, and other reducing biomolecules that can participate in the reduction of Ag+ and aid in the stabilization of the nanoparticle surface.

Thus, in this study, the extract of *Origanum dubium* was used as a biological reducing and capping system. This method reduces the necessity of externally added chemical reducing agents and offers a relatively simple synthetic route while still permitting the optimization of nanoparticle formation through control of the pH, temperature, precursor concentration, extract concentration, reaction time, and irradiation conditions. Two bacterial strains, the Gram-positive methicillin-resistant *Staphylococcus aureus* (MRSA) and the Gram-negative *Escherichia coli* (*E. coli*), were used to test the antibacterial properties of the synthesized AgNPs, MWCNTs, functionalized MWCNTs, and their AgNP-decorated nanocomposite forms. The optical, chemical, structural, and morphological characteristics of the pristine and functionalized MWCNTs and their AgNP-decorated nanocomposites were examined using UV–vis spectroscopy, Fourier-transform infrared spectroscopy (FTIR), X-ray diffraction (XRD), and scanning electron microscopy (SEM). Considering the reported reducing and stabilizing capacities of phytochemicals of plant origin, we hypothesized that *Origanum dubium* extract could be a natural reducing and capping system for the formation of AgNPs under optimized synthesis conditions and, at the same time, could assist their deposition on pristine and acid-functionalized MWCNT surfaces. Furthermore, we hypothesized that the optimization of parameters, including temperature, AgNO_3_ concentration, plant extract concentration, pH, reaction time, and irradiation time, would affect the optical and colloidal properties of the generated AgNPs and that incorporation of AgNPs onto MWCNTs, especially functionalized MWCNTs, would enhance the antimicrobial and antioxidant activities of the produced nanocomposites as compared to the individual components.

## 2. Results and Discussion

### 2.1. The Phytochemical Composition of O. dubium Extract

Mixtures of volatile and semi-volatile substances from organic or biological sources can be identified, quantified, or separated using gas chromatography/mass spectrometry (GC–MS), a hyphenated analytical technique [40]. The methanolic leaf extract of *O. dubium* has been subjected to bio-constituent analysis using gas chromatography-mass spectrophotometry (GC-MS). *O. dubium* leaf extracts contain a number of bioactive elements with a range of biological activities, according to the results (Table 1), with retention time (Ret. Time), molecular formulas, and the type of compounds. The methanolic extract contained p-Cymene-2,5-diol, Thymoquinone, Lidocaine, Phenol, 2,2’-methylenebis [6-(1,1-dimethylethyl)-4-methyl-, 3-Methyl-4-isopropylphenol, 2,4-Di-tert-butyl phenol, 4-Cyanophenol, and Benzyl alcohol. Origanum species such as *Origanum dubium* have a bioactive terpenoid called p-Cymene-2,5-diol, sometimes referred to as thymohydroquinone. By rupturing the cell membranes of pathogens like *Escherichia coli* and *Staphylococcus aureus*, it demonstrates potent antibacterial qualities and functions as a natural antioxidant [41]. 3-Methyl-4-isopropylphenol, sometimes referred to as o-cymen-5-ol or IPMP, is an artificial or naturally occurring monoterpenoid phenol isomer of carvacrol and thymol. Carvacrol and other phenolic monoterpenes are abundant in extracts from *Origanum dubium* (Cypriot oregano). Carvacrol has potent antioxidant qualities against vitamin E, butyl hydroxyl toluene (BHT), and ascorbic acid. Carvacrol has been shown to have anti-proliferative effects on non-small lung cancer cells, A549, chronic myeloid leukemia cells, K562, and murine B16 melanoma cells; however, the molecular mechanisms underlying these effects are yet unknown [42]. In addition to non-volatile components, carvacrol and other volatile terpenes, such as carvacrol acetate and spathulenol, can occasionally be extracted and identified. But once carvacrol is in the extracts, it may have pharmacological effects [43].

### 2.2. Effect of Temperature on the Synthesis of AgNPs

**Figure 1:**
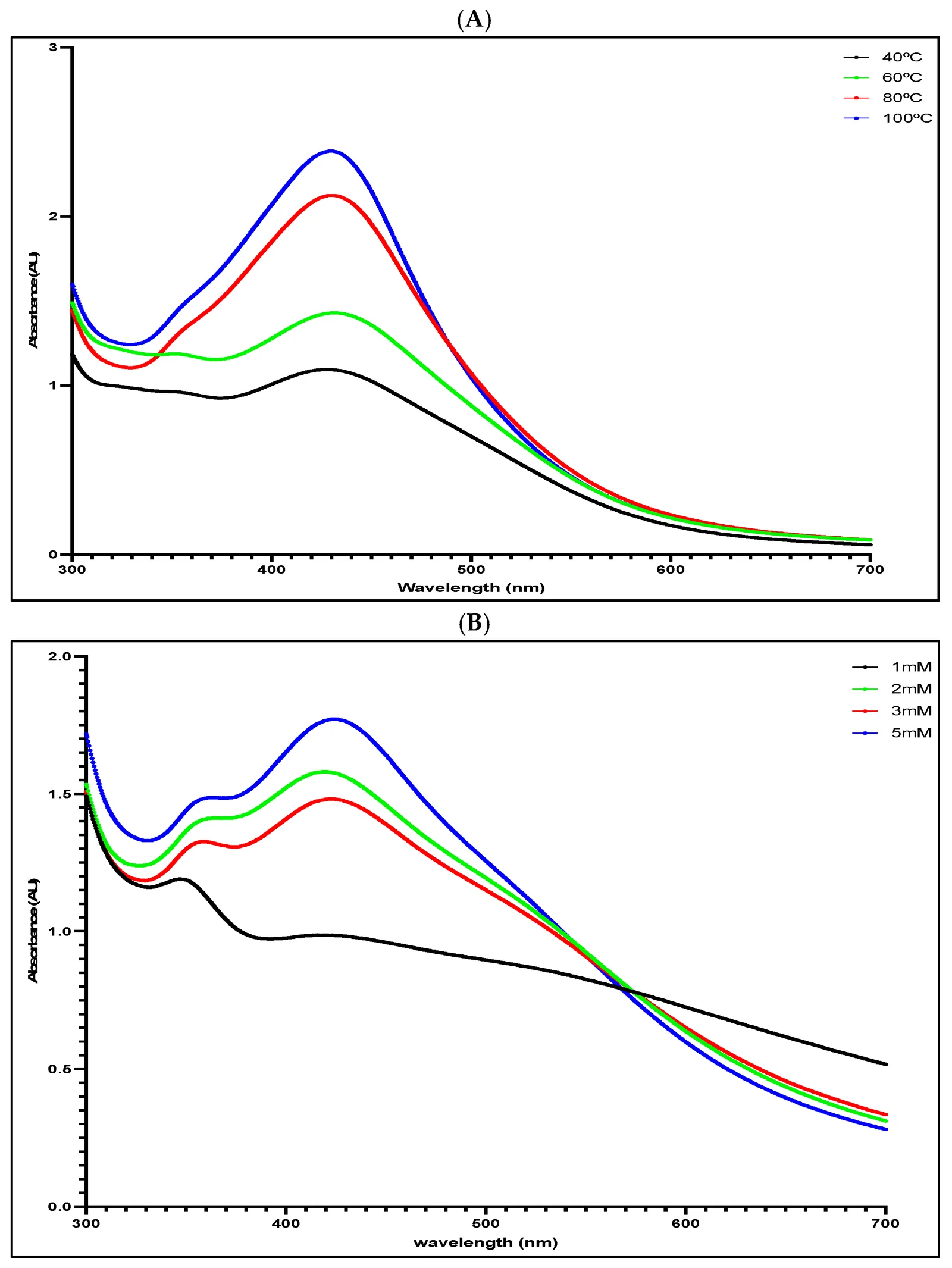

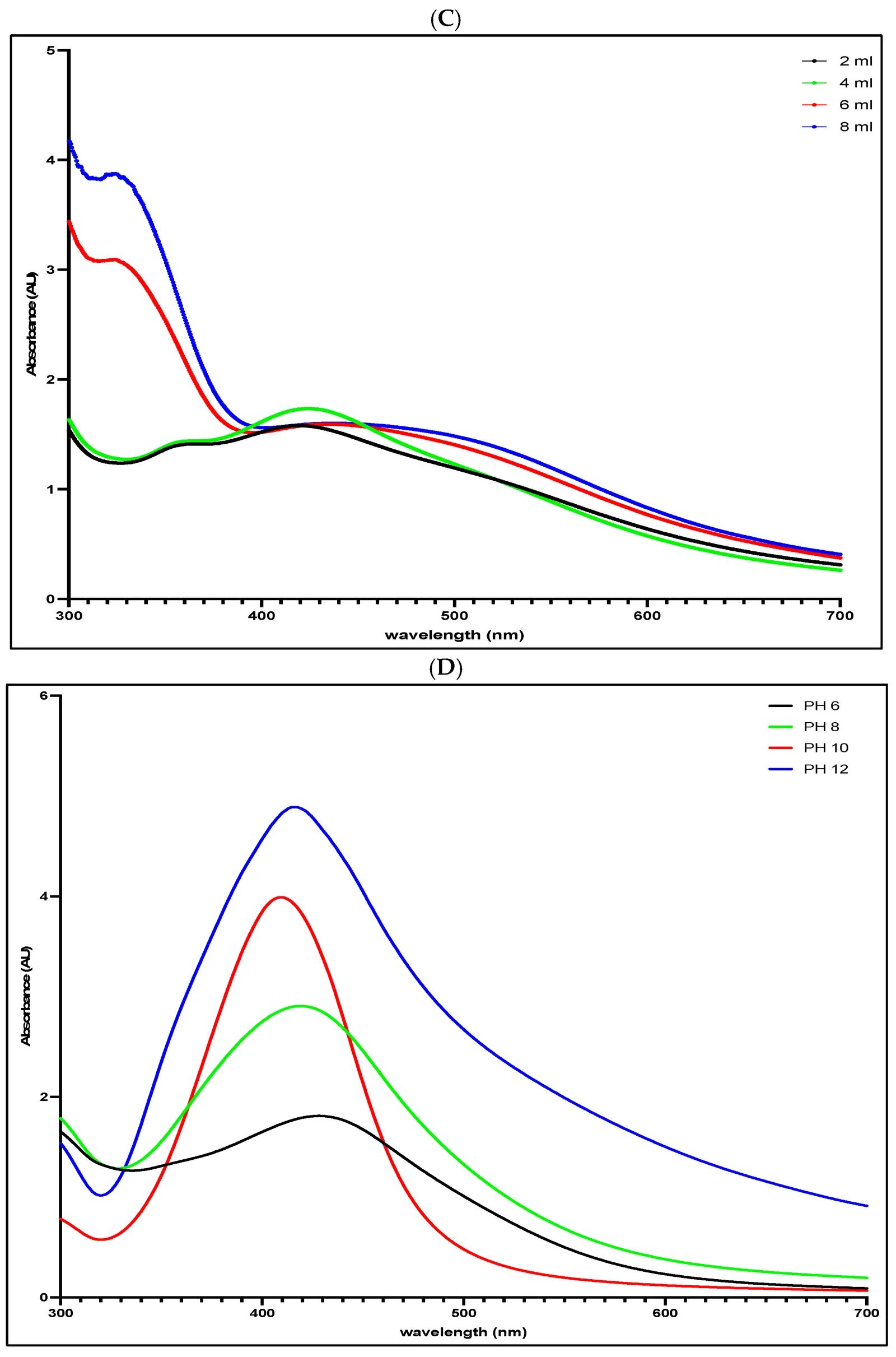

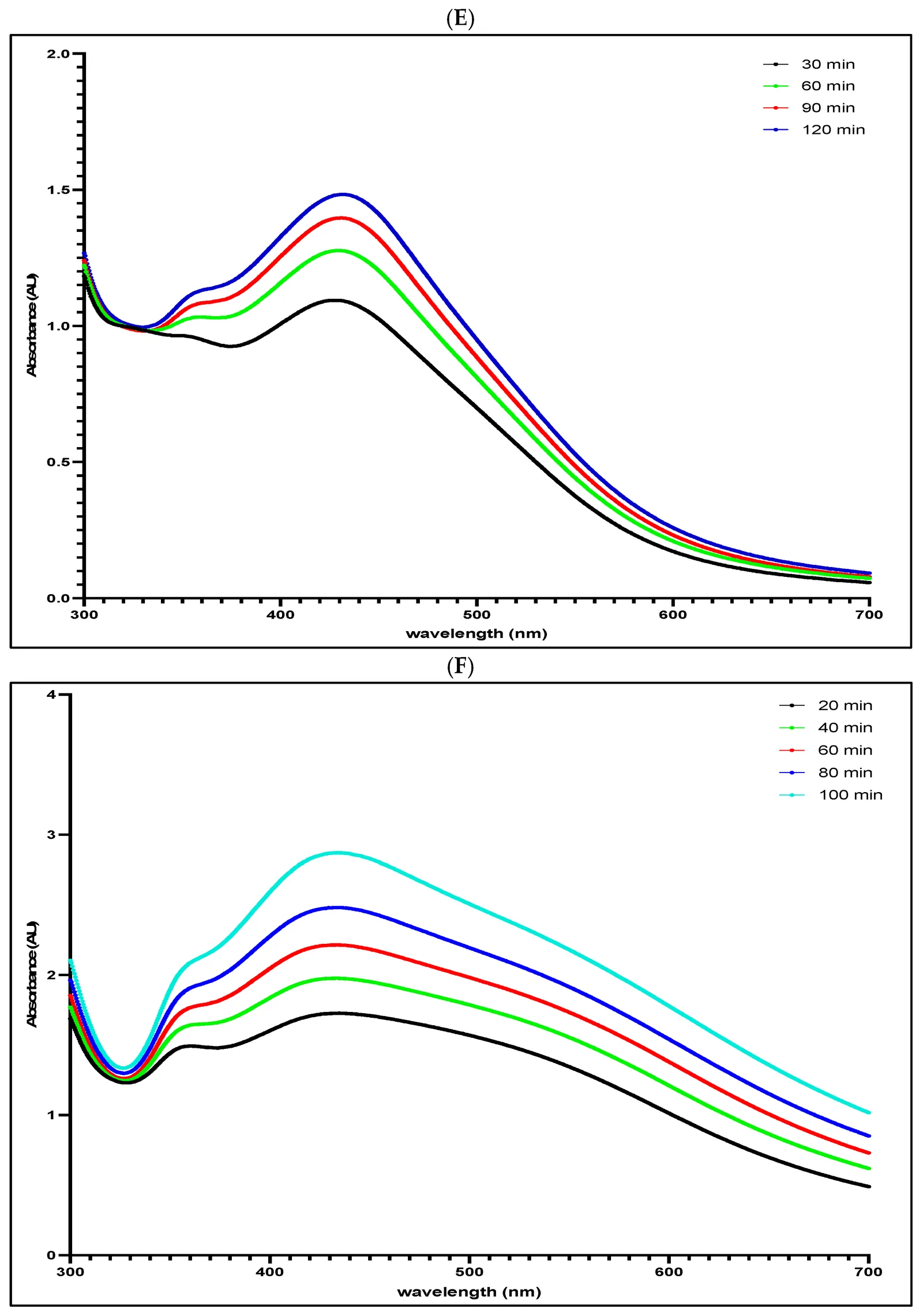
UV–vis spectra illustrating the optimization of AgNP synthesis using *Origanum dubium* extract: (**A**) effect of reaction temperature; (**B**) effect of AgNO_3_ precursor concentration; (**C**) effect of plant extract concentration; (**D**) effect of reaction pH; (**E**) effect of reaction time; and (**F**) effect of irradiation time.

According to the results, the localized surface plasmon resonance (LSPR) is responsible for the absorption band that appears at around 430 nm when the produced AgNPs are present. The strength of this band is directly influenced by the concentration of AgNPs in the colloid. Therefore, variations in the strength of the SPR band may be clearly attributed to the reaction temperature, as seen in Figure 1A. The size development of AgNPs may be monitored using UV spectroscopy, which detects changes in the surface plasmon resonance bands shown at various wavelengths and absorption intensities. The rapid creation of AgNPs was seen at the maximum tested temperature of 100 °C, accompanied by an increase in the intensity of surface plasmon resonance absorption with time, as shown in Figure 1E. Increasing the temperature from 40 °C to 100 °C caused a red-shift in the local maxima of the absorption bands. Therefore, at higher temperatures, the size of the AgNPs increased, as evident from the shifted SPR peaks. This shift was attributed to thermal instability of the nanoparticles, as the particles tend to agglomerate due to increased surface energy [44,45]. Therefore, the optimal temperature was considered to be 40 °C for the formation of small-size nanoparticles that had relatively higher colloidal stability than the ones synthesized at higher temperature ranges.

Typically, the production of metal nanoparticles involves multiple mechanistic processes. The process begins with the reduction of the metal precursor and the subsequent production of metal atoms. This is then followed by the aggregation of metal monomers into dimers and primary clusters [46]. The merging and spreading of metal particles on the surface of the particle result in the enlargement of initial clusters. The reduction of the metal precursor happens at a relatively moderate pace, which can limit the growth of these nanoparticles due to this reaction [47].

Strong reducing agents accelerate the reduction of metal ions and the production of primary clusters, resulting in faster reaction rates. This leads to improved growth of primary particles due to their coalescence, which occurs soon after the addition of reagents. The rate at which nanoparticles grow when they merge together is determined by their ability to surpass the kinetic barrier, also known as the activation energy. This barrier arises from the electrostatic repulsion between the charged surfaces, which results in the formation of electrical double layers [48]. In addition, the stabilizing chemicals have the potential to adsorb onto the surface of the charged nanoparticles, which can impede their growth and aggregation by providing steric stabilization [49].

### 2.3. Effect of Silver Nitrate Concentration on the Synthesis of AgNPs

The color of the reaction mixture transitioned from pale yellow to dark orange, with the intensity of the color deepening in proportion to the concentration, indicating a higher concentration of AgNPs. Figure 1B,F depicts the UV–vis absorption spectra of the reaction mixtures, which were obtained at 5 min intervals between 300 and 700 nm. The concentration of precursors is a critical factor in nanoparticle formation [27]. The size and shape of nanoparticles produced are significantly affected by the amount of AgNO_3_. The study found that as the concentration of silver nitrate solution increased, the production of AgNPs obtained from methanolic plant extract was improved (Figure 1B). The absorption spectra exhibited a redshift with increasing concentrations of silver nitrate precursor, suggesting the creation of large nanoparticles. The increase in size of nanoparticles is clearly observed at great concentrations of AgNO_3_, as indicated by the shift in the absorption spectra towards longer wavelengths [44].

### 2.4. Effect of Plant Extract Concentration on the Synthesis of AgNPs

The quantity of plant biomass is crucial in the process of reducing silver salt. It was observed that the concentration of nanoparticles in the colloid decreased when the concentration of methanolic plant extract exceeded 4 mL. The SPR peaks migrated towards longer wavelengths, spanning a wider range of the UV region (Figure 1C). This indicates the formation of large-sized nanoparticles as well as increased variation in the size of the AgNPs owing to a higher polydispersity index [27]. Regarding the methanolic AgNPs, as the concentration of biomass increased from 2 mL to 4 mL, the peak intensity in the 420–430 region increased in absorption, suggesting an increased rate of reduction of silver ions and thus the nucleation process. Beyond 4 mL of the methanolic plant extract, the SPR peak intensities declined, in addition to a red shift to longer wavelengths, indicating the large amount of nanoparticles synthesized. In addition, the bandwidth of the SPR peaks was recorded to be broader in direct proportionality to the increasing leaf extract quantity, exhibiting the formation of a large range of sizes in the distribution of nanoparticles [18]. The methanolic plant extract has excellent capacity for reducing silver salt at 4 mL as evident from the elevated SPR peak absorption intensity, beyond which the size of the nanoparticles was recorded to increase. Hence, 4 mL was chosen as the optimum plant extract biomass for effective synthesis of AgNPs.

### 2.5. Effect of pH on the Synthesis of AgNPs

The pH of the reaction is another crucial element in the synthesis of AgNPs. By changing the electrical charges of the phytoconstituents, the pH of the solution may affect the AgNPs’ size and structure. Consequently, this has an impact on the characteristics of the capping agents and the stability of the AgNPs. Figure 1D depicts the relationship between the pH of the reaction mixture and the rate of synthesis.

It clearly shows that as the pH increases from 6 to 12, the synthesis rate becomes faster. Throughout the pH range tested, there is a clear link between the rising levels of hydroxide ions in the system and the concentration of synthesized AgNPs. This is corroborated by the existence of prominent absorption peaks within the wavelength range of 411 to 426 nm. Elevated pH values have been found to increase the bioavailability of phytoconstituents, resulting in an accelerated pace of response. This is apparent from the rapid change in hue noticed in the reaction mixture. Moreover, it has been shown that using an alkaline pH during the synthesis of AgNPs might result in several benefits. These benefits consist of improved stability and a greater production of nanoparticles, as shown by the heightened SPR peak, accelerated growth rate, and effective reduction process of Ag+ to Ag0.19, 23. The cause of this phenomenon may be attributed to the elevated quantity of hydroxyl (OH) groups present in plant extracts. The hydroxyl (OH) groups have a vital function in decreasing and securing functional groups when AgNPs are being formed. Research has shown that when the pH level rises, the number of -OH groups participating in the reduction process also increases, leading to a great reduction yield [23]. The increased formation of AgNPs in an alkaline environment should not be considered an increase in the absolute number of hydroxyl groups in the plant extract. Instead, increasing the pH may alter the protonation state of hydroxyl-containing phytochemicals, especially phenolic compounds, resulting in an increased fraction of the deprotonated species [31,34]. Such deprotonation may enhance the electron-donating ability of suitable phytochemicals and thereby facilitate the reduction of Ag+ to Ag0. Alkaline conditions can also modify the coordination and adsorption of phytochemical molecules onto the surface of the nanoparticle, influencing nucleation, growth, and colloidal stability. In contrast, at more acidic conditions, more of the protonatable functional groups are protonated, and this can lead to a reduced availability of some phenolate-type electron-donating species. The observed pH dependence is therefore probably due to changes in phytochemical ionization and reaction kinetics rather than to the formation of additional hydroxyl groups [17,18,19].

The shift in the SPR peaks towards shorter wavelengths from pH 6 to 10 indicates the presence of small AgNPs. Additionally, the narrowing of the UV region under alkaline conditions suggests that the solution is monodisperse, with consistent nanoparticle sizes. Nevertheless, it can be reasonably inferred that the alkalinity exceeding a pH of 10 for the methanolic AgNPs contributes to the increased polydispersity of the AgNP colloid. This is demonstrated by the large-sized nanoparticles and the expanding SPR peak, which are caused by the absorption band’s redshift towards a longer wavelength range [23]. Similar to the methanolic AgNPs, when the alkalinity exceeded a pH of 10, a distorted peak with a decreased intensity of the SPR band in the 410–430 nm region was observed. Hence, it was determined that a pH of 10 is the most suitable for the synthesis of AgNPs using methanolic extract. This suggests that the resulting AgNPs are small in size, have a spherical shape, and are evenly dispersed.

The pH optimization experiment was constrained in that the pH was adjusted by direct addition of NaOH, rather than a buffered system. This approach avoided the introduction of potentially interacting buffer species into the Ag+-phytochemical reaction system, but increasing NaOH also altered the ionic composition and ionic strength of the reaction medium. Thus, the observed variation in AgNP formation with pH may be ascribed to the synergistic effect of alkalinity, ionic strength, and changes in the ionization state of the phytochemical constituents. Well-chosen buffer systems in future studies or experiments conducted under controlled ionic strength would help to distinguish these effects.

### 2.6. Effect of Reaction Time on the Synthesis of AgNPs

The reaction time is important for the synthesis of AgNPs. The reaction mixture consisted of 2mM AgNO_3_, 2 mL extract, pH 6, and a temperature of 40 °C (Figure 1E). The color of the methanolic extract solution changed from pale yellow to dark orange, and further intensified to a dark brown as the reaction progressed, indicating the successful synthesis of AgNPs.

### 2.7. Effect of UV-Irradiation on the Synthesis of AgNPs

Modifying the exposure to UV radiation is another way to optimize the creation of nanoparticles. As the duration of UV exposure increased, so did the SPR peak height. The SPR peak has blue-shifted to a lower wavelength after being incubated for 20 to 80 min, suggesting the creation of smaller nanoparticles. Beyond 80 min, a red-shift is observed. Beyond 20 min of UV irradiation, the SPR peak shifts to a longer wavelength from 430 to 440.5 nm as the reaction proceeds. The largest shift occurs between 40 and 60 min, implying the formation of large-sized nanoparticles [50,51]. Therefore, the optimum UV-light exposure time was determined to be 60 min for the methanolic AgNPs.

The synthesis parameters investigated here were considered as interrelated variables that affected the reduction of Ag+ ions, nucleation, particle growth, and colloidal stability. The reaction kinetics and particle expansion were mainly affected by temperature, and the presence of Ag+ ions as a precursor was dictated by the concentration of AgNO_3_. The amount of *O. dubium* extract influenced the levels of phytochemical reducing and stabilizing agents in the synthesis of AgNP. In addition, pH altered the ionization state and reducing potential of phytochemical constituents, thus affecting the reduction rates and properties of particles [28,29]. The development and finalization of nanoparticle production were also influenced by the rate of reaction and exposure to sunlight. In this way, the parameters were studied individually through UV–vis observation, and the conditions related to the most favorable SPR properties were selected for the next development of the AgNP/MWCNT nanocomposites. The optimal synthesis was carried out under the stated conditions using the plant extract-mediated green approach.

### 2.8. UV–Vis Spectra of Nanomaterials

**Figure 2:**
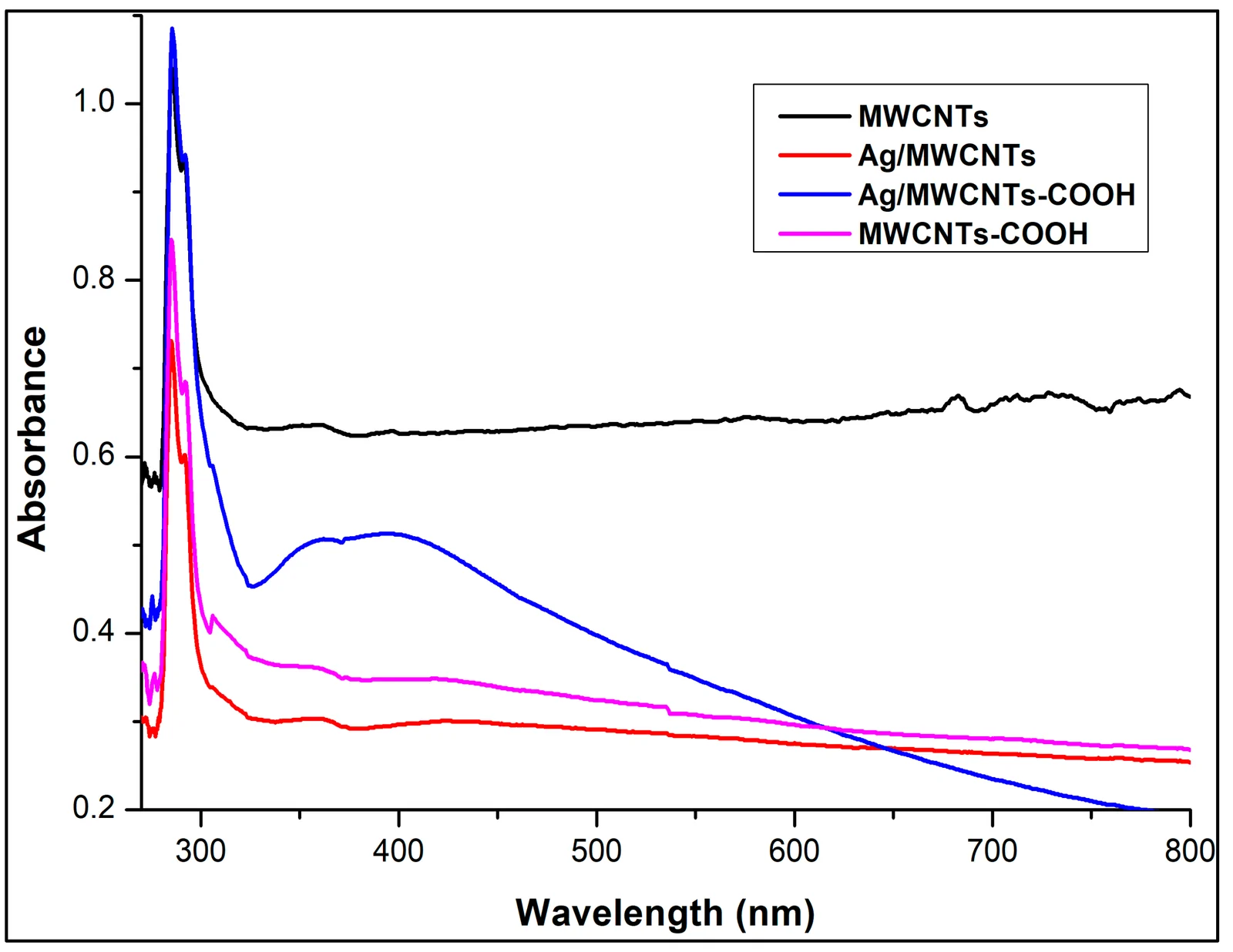
UV–vis spectra of pristine multi-walled carbon nanotubes (MWCNTs), COOH-functionalized MWCNTs, Ag/MWCNTs, and Ag/MWCNT-COOH nanohybrids.

The UV–vis spectra of functionalized MWCNTs, pristine MWCNTs, and their AgNPs nanohybrids are shown in Figure 2. The pristine MWCNTs exhibited an absorption band in the region of 280–300 nm, showing a decrease in absorptivity. This is a characteristic behavior seen in MWCNTs. After acid treatment under reflux at high temperatures, the UV–vis of the f-MWCNT does not demonstrate distinguishing absorption that would set the difference between the pristine and surface-modified MWCNTs. The characteristic maximum absorption in the 290–300 nm range is present, but no shift in the characteristic band is observed due to the hydroxyl and carboxyl groups, which is in contrast with the previous literature [20,21,22]. To account for this negligible electronic transition in the case of f-MWCNT, this study hypothesizes that the carboxylic acid functionalization process may not have been efficient enough to result in such a concentration of COOH groups on the MWCNTs that is detectable by the electronic transition properties analyzed by UV–vis spectroscopy. Therefore, the expected changes in electronic structure and absorption properties may not have been significant.

Alternatively, the UV–vis absorption of pristine MWCNTs is often attributed to π-π* transitions, which are affected by the electronic structure and arrangement of π-bonds [50]. Similarly, if the functionalization process during acid treatment was not effectively adequate in altering the π-electron system, the absorption may remain largely unaffected in the UV–vis spectrum. Pristine MWCNTs are often attributed to π-π* transitions, which are affected by the electronic structure and arrangements of π-bonds. Similarly, if the functionalization process during acid treatment was not effectively adequate in altering the π-electron system, the absorption may remain largely unaffected in the UV–vis spectrum [52,53].

The UV–vis absorption spectra of AgNPs and Ag/MWCNT nanocomposites were examined to clarify the optical properties and potential interactions between silver nanoparticles and carbon nanotubes.

An absorption band seen at around 400 nm in the samples containing AgNPs is ascribed to the localized surface plasmon resonance (LSPR) of silver nanoparticles. This plasmonic characteristic results from the collective oscillation of conduction band electrons in reaction to incoming electromagnetic radiation. The position and width of this band are significantly affected by the distribution of particle sizes, their morphology, and the dielectric medium around the nanoparticles [23,24,25].

In the case of the Ag/MWCNT nanocomposite, the plasmon band exhibits a wider profile in comparison to AgNPs alone. This expansion may correlate with enhanced size dispersion, partial aggregation, and, crucially, electronic interaction between AgNPs and the MWCNT surface, potentially resulting in plasmon damping effects.

The absorption pattern observed at around 250 nm is ascribed to π–π* interband transitions of the C=C bonds in the graphitic structure of MWCNTs. These transitions are indicative of sp^2^-hybridized carbon systems and validate the existence of the nanotube structure.

The optical response of the composite system may involve interband transitions in silver, contributing to the total absorption background and potentially overlapping with the plasmon resonance, thereby affecting peak form and intensity.

The interaction between AgNPs and functionalized MWCNTs may enhance charge transfer processes at the interface, hence altering the local electron density and influencing plasmonic activity. Such interactions are recognized to modify both the strength and breadth of the plasmon band.

The obtained spectral characteristics imply the successful integration of AgNPs onto the MWCNT surface and suggest potential electrical interactions between the two components.

As a result, the AgNPs attached to the surface of the modified MWCNTs with reduced clumping, resulting in a decrease in polydispersity [54,55,56]. Additionally, the strong binding of the AgNPs to the surface of the MWCNTs was facilitated by the layer of reducing agents surrounding the AgNPs [57,58].

### 2.9. Fourier Transform Infrared Spectroscopy (FT-IR)

**Figure 3:**
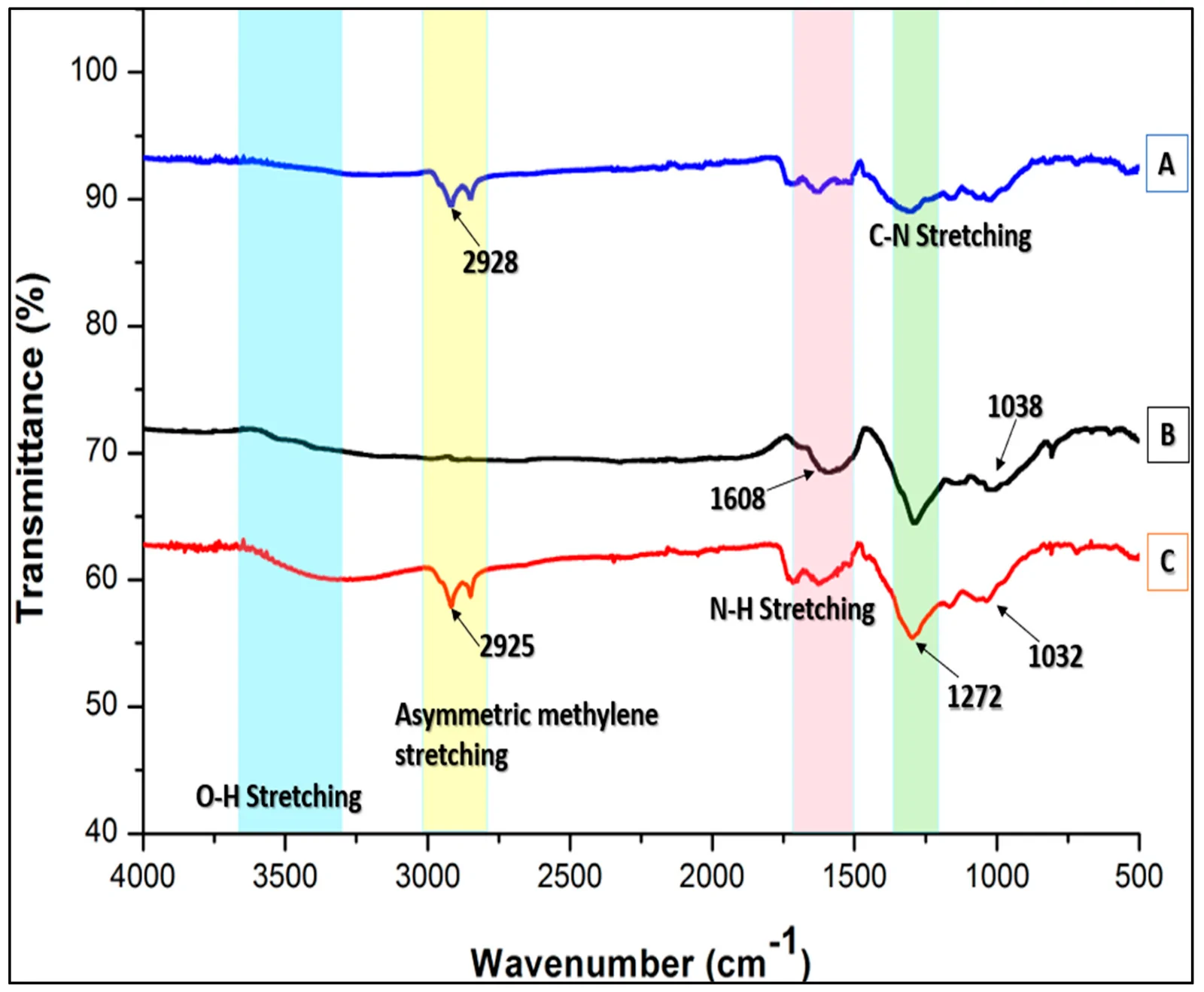
FTIR spectrum of (A) pristine-MWCNTs, (B) COOH-functionalized MWCNTs, and (C) AgNP-decorated surface-functionalized MWCNTs.

**Figure 4:**
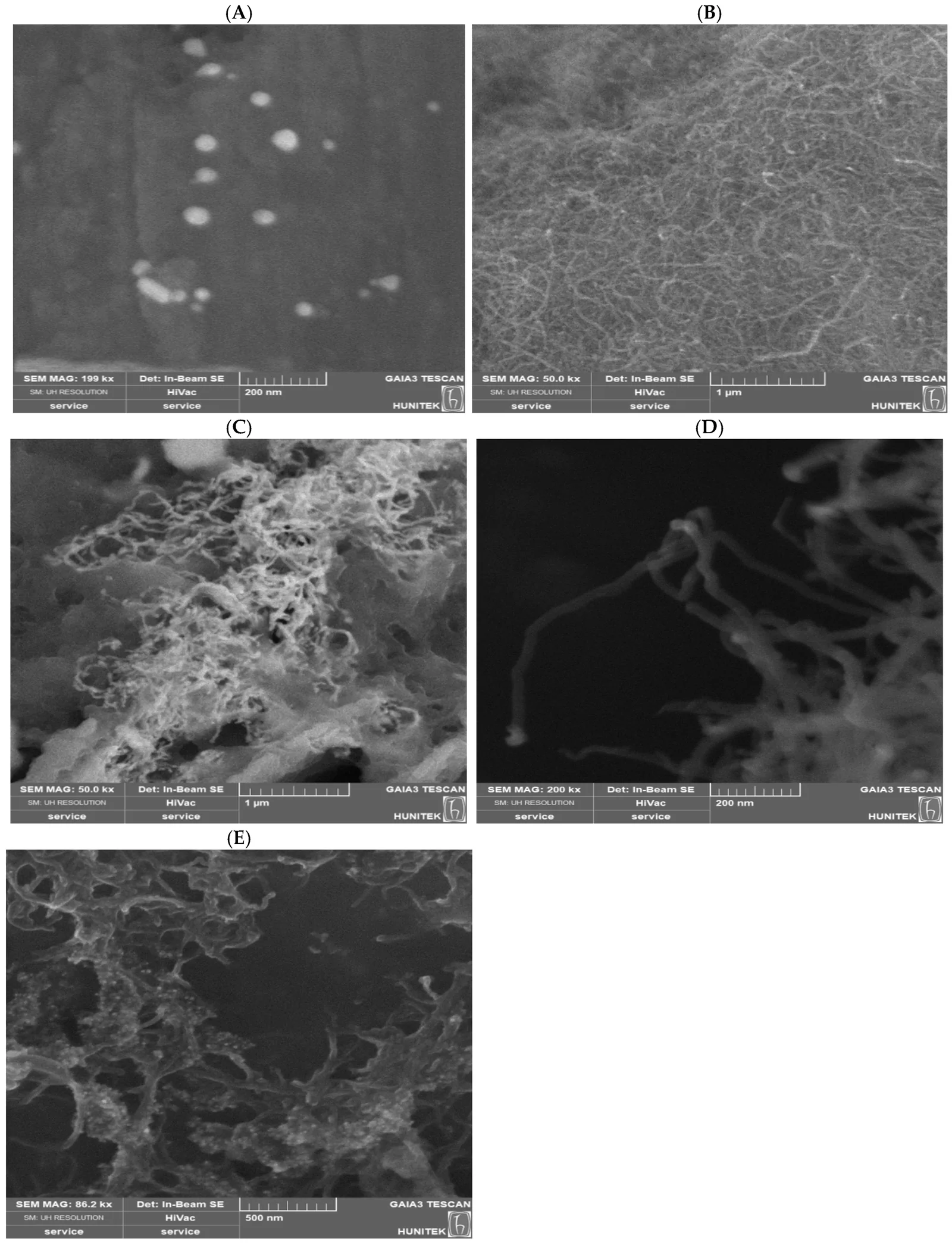
SEM images of (**A**) silver nanoparticles prepared in methanol, (**B**) pristine MWCNTs, (**C**) surface-functionalized nanostructures, (**D**) AgNP-decorated pristine MWCNTs, and (**E**) MWCNT-COOH with AgNPs deposited on them.

The FTIR spectra of pristine MWCNTs, functionalized MWCNTs, and their decorated nanoforms were compared. It was observed that surface-functionalized MWCNTs (B) exhibited an absorption band at 3300–3600 cm^−1^, which corresponds to the O-H stretching vibration shown in Figure 3. The C=C bonding of the carbon skeleton structure comprising the nanotubes was observed at 1600 cm^−1^. The N-H stretch at 1608 cm^−1^ in the amide links acts as a stabilizing and capping agent, as mentioned in previous studies [59,60,61]. The peaks at 1038 cm^−1^ and 1022 cm^−1^ in Figure 4B and Figure 4C, respectively, show slight variation in the case of AgNPs-decorated MWCNTs. This suggests that silver ions and functional groups are interacting, either by simple electrostatic attraction or via the creation of a coordination bond [59]. Furthermore, the absence of certain vibration bands in pMWCNTs (Figure 4A) and functionalized MWCNTs, specifically at 2928 cm^−1^ and 2925 cm^−1^, confirms the involvement of various functional groups in the bio-reduction mechanism. This ultimately leads to the biosynthesis of AgNPs/MWCNT-COOH, as depicted in Figure 3A and Figure 3C, respectively. The FTIR analysis suggests that the AgNPs are surrounded by terpenoid, alcohol, and carbonyl groups, which act as binding sites for AgNPs [60].

### 2.10. Scanning Electron Microscopy

The surface morphology of MWCNTs was examined using SEM before and after treatment with nitric and sulfuric acid under reflux conditions. The SEM image is shown in Figure 4. No structural damage was observed in MWCNTs that were functionalized with polar functional groups. On the other hand, homogenous distribution of AgNPs without agglomeration was observed adhering to the surface-functionalized MWCNT hybrid, corroborating the effect of oxygen-containing functional groups on the formation of Ag/MWCNT-COOH nanocomposites. Previous studies have shown that Tollens reagent or DMF are often used chemicals to reduce silver salts for the formation of AgNPs. This process allows for the production of stable colloids with tiny diameters and stable aqueous dispersions [61].

The objective of the surface modification was to increase the number of active sites on the surface of the MWCNTs for the binding of AgNPs. This was achieved using a methanolic plant extract, which is a green method, instead of using toxic reductants like Tollens and DMF. The SEM images also reveal the roughly flower-shape homogenous structures of green-synthesized AgNPs. The methanolic AgNPs prepared using plant extract showed a very porous and fibrous structure with clear and visible edges. Interestingly, methanol as a solvent was able to extract phytochemicals that could result in the formation of monodisperse and homogenous silver nanostructures composed of well-assembled and loosely packed AgNPs [62].

### 2.11. Size Distribution and Zeta Potential

Zeta potential is a method used to analyze the electric potential in the immediate vicinity of a particle that is suspended in a liquid medium. The repulsive interactions between particles are responsible for this phenomenon and may be used to evaluate the stability of particle dispersion. From Table 2, the nanohybrid composed of functionalized MWCNT support decorated with AgNPs showed the highest degree of stability, with a zeta potential amounting to −38.93 mV, posing a negligible potential for agglomeration. Pristine MWCNTs decorated with green-synthesized AgNPs had a zeta potential of −21.3 mV, which is high enough to minimize the risk of aggregation according to the accepted parameters of stability. The presence of polar functional groups, such as carboxyl and hydroxyl groups, on the surface of the functionalized MWCNTs is responsible for the reduction in colloid stability of the nanohybrids. Furthermore, this increase acts as a nucleation site for the AgNPs that will later be deposited on the nanotube surface [59]. As a result, MWCNT-COOH and its nanohybrid structure are more stably dispersed inside the solution, improving a uniform distribution as evident from lower polydispersity indices, 0.1419 and 0.3626, respectively. On the other hand, when the zeta potential falls outside the standard limit for achieving significant stability, −30 mV, the van der Waals forces promoting attraction exceed repulsion, thus leading to the formation of aggregates, such as the pristine MWCNTs with higher zeta potential values and a size of 230.9 nm. When metals such as silver are deposited onto the MWCNTs, the zeta potential experiences an improvement in terms of colloid stability from −12.18 to −21.3 mV. Additionally, the size of the Ag/MWCNT hybrid was recorded to be smaller as the forces of attraction between them were minimized. One notable characteristic of the Ag/MWCNT nanohybrid was that the zeta potential value slightly increased from −22.58 to −21.3 mV.

### 2.12. XRD (X-Ray Diffraction) Assay

**Figure 5:**
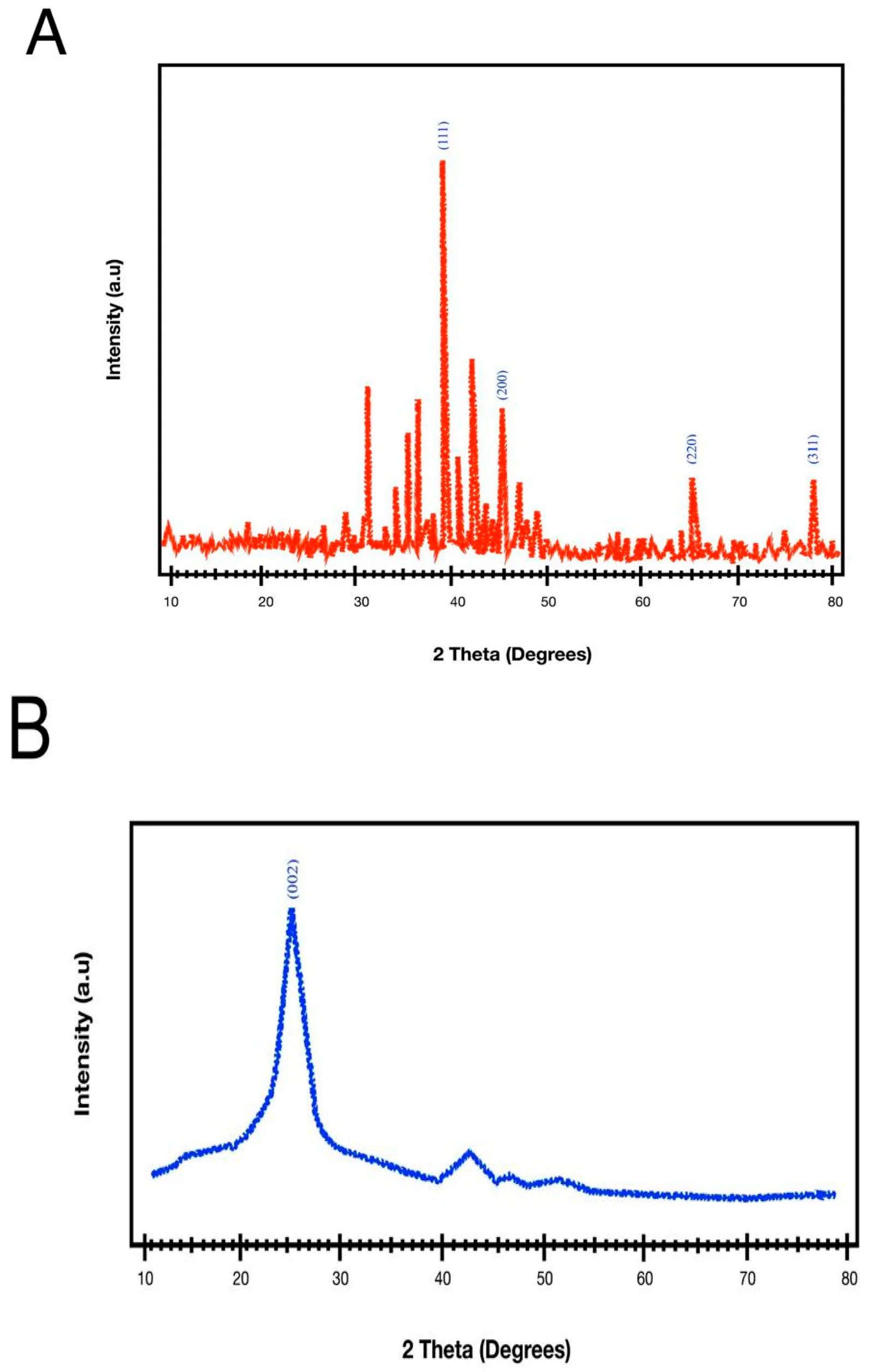

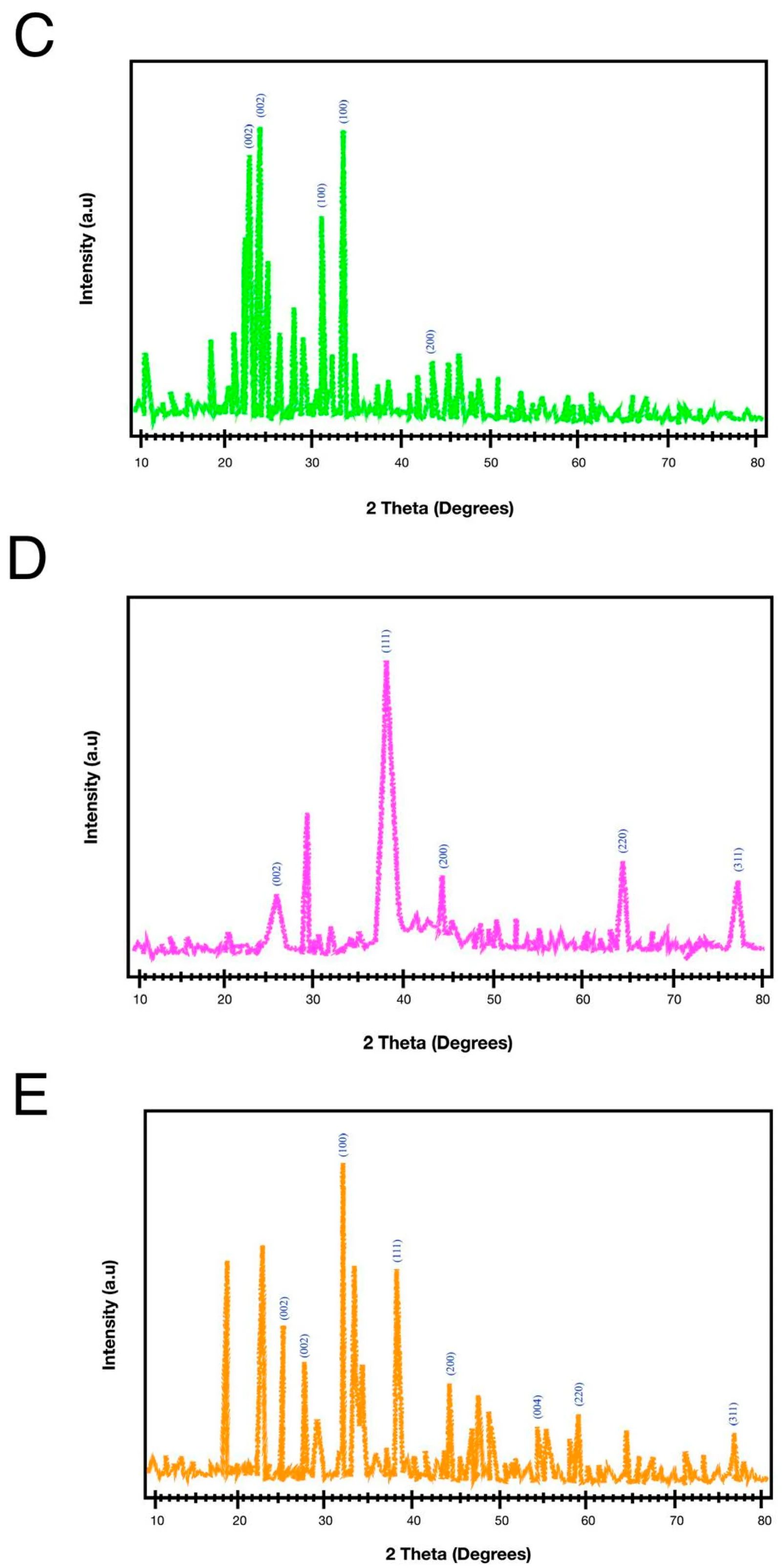
XRD analysis of (**A**) biosynthesized AgNPs, (**B**) MWCNTs with pristine surface, (**C**) surface-functionalized MWCNTs with polar hydroxyl functional groups, (**D**) AgNP-deposited pristine MWCNTs, and (**E**) functionalized MWCNTs decorated with AgNPs.

As seen in Figure 5A, the XRD patterns were obtained for the AgNPs synthesized from *O. dubium*. The intense peaks of AgNPs at 31.1, 32.98, 34.16, 35.2, 37.86, 42.76, 43.84, 44.32, 63.96, 64.18, and 77.54 appeared to be indexed as crystalline silver. Presumably, the development of spherical AgNPs was suggested by the peaks’ sharpness. The Debye–Scherrer formula [61] was used to determine the average size of the nanoparticles. The XRD pattern of f-MWCNT-Ag nanohybrids is shown in Figure 5E, revealing different diffraction peaks at certain angles: 18.98°, 23.12°, 25.02°, 27.98°, 32.04°, 38.04°, 44.2°, 47.28°, 55.32°, 59.42°, 64.34°, and 77.3°. The distinct diffraction peaks typically seen in functionalized MWCNTs are obscured in surface-modified MWCNTs that have been coated with AgNPs. This is because the AgNPs, which are tightly bound to the surface of the functionalized MWCNTs, produce very intense peaks as a result of their metallic state [45,52]. The XRD patterns of surface-functionalized MWCNTs are shown in Figure 5C. The average crystallite size of the Ag phase was estimated from the major AgNPs reflection using the Scherrer equation. The obtained crystallite sizes were 14.76 nm for AgNPs, 18.24 nm for Ag/MWCNTs, and 25.22 nm for Ag/MWCNT-COOH, calculated from the fitted full width at half maximum (FWHM) values. The XRD pattern of Ag/MWCNTs was also characterized by the typical MWCNT-related reflection at about 26° and the Ag reflections at about 38°, 44°, 64°, and 77°. The Ag/MWCNT-COOH sample showed the characteristic Ag reflections along with support-related reflections attributed to functionalized MWCNT material.

MWCNT-COOH in the range 10–80° (2θ) demonstrated some small noise-like peaks beyond 40°, and two main peaks corresponding to 26.26° and 43.12°. The crystalline structure of the AgNP-deposited pristine MWCNTs was assessed by XRD analysis. The diffraction peaks with 2θ value of 26.18°, 29.3°, 38.04°, 44.1°, 64.38°, 77.36°. The decrease in angle at 26.18° is caused by an expansion in the distance between the sp^2^ C=C layers, when compared to AgNPs that are biosynthesized. The XRD findings indicate that the composites consisting of Ag and MWCNTs have crystalline structures and show a clear distribution of AgNPs on the MWCNTs [62].

### 2.13. Antimicrobial Activity Using Well Diffusion Assay

**Figure 6:**
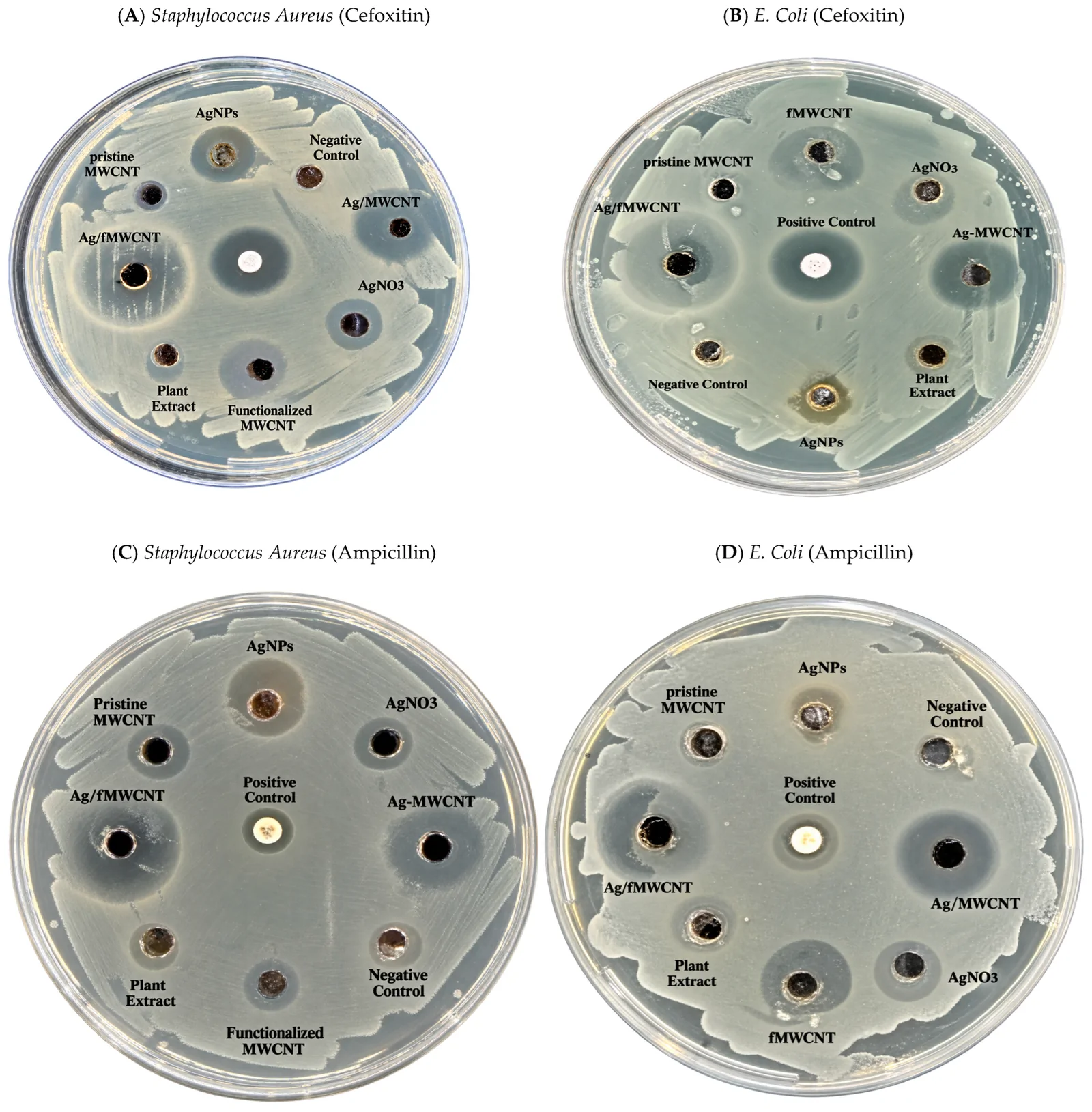
Inhibition zones of 200 µg/mL of AgNP, plant extract, pristine and functionalized MWCNTs, and their nanohybrid, decorated structures implanted on Gram-positive *Staphylococcus aureus* (MRSA) and *Escherichia coli*. (**A**) *S. aureus* with cefoxitin; (**B**) *E. coli* with cefoxitin; (**C**) *S. aureus* with ampicillin; and (**D**) *E. coli* with ampicillin.

AgNPs and the prepared nanocomposites of Ag/MWCNTs exhibited high antimicrobial activity due to the effect of Ag in terms of the zone of inhibition compared to aqueous plant extract, as shown in Figure 6. Clearly, the AgNPs, functionalized MWCNTs, and decorated f-MWCNTs have notable antibacterial characteristics based on the measured diameter of the zone of inhibition, which are 0.529 cm, 1.107 cm, and 1.714 cm, respectively. These results are reported in Table 3. The findings indicate that the Ag/MWCNTs, with both the pristine and functionalized support systems, exhibit improved antibacterial properties (1.393 and 1.714 cm, respectively) compared to the AgNPs acting as standalone antimicrobial agents. This heightened antimicrobial effect can be highly attributable to the MWCNTs. AgNPs and other metal oxides have been demonstrated in several studies to possess antibacterial qualities and the capacity to eradicate microorganisms that cause diseases. Overall, studies have demonstrated that AgNPs have the ability to penetrate bacterial cell membranes and alter their structural makeup, which ultimately causes the cell to die [62,63].

Therefore, the combined synergistic effect of the AgNPs and the MWCNTs increased the toxicity of the nanotubes [24,29]. The presence of hydroxyl and carboxyl moieties in MWCNTs nanocomposites led to increased antibacterial activity. This is attributed to the formation of CNT-bacterial aggregates, which enhance the interactions between the nanocomposites and the bacteria [23]. The results are consistent with previous research that found that functionalizing CNTs enhances their binding with bacterial cells [61,63]. The antibacterial activity of AgNPs was more pronounced against the Gram-positive methicillin-resistant *Staphylococcus aureus* (MRSA) strain. The variation in cell wall composition accounts for this distinction. Silver ions from nanoparticles are drawn to the negatively charged bacterial cell wall. Once drawn to the cell wall, they adhere to it, changing its composition and permeability in the process [64]. When bacteria are exposed to silver ions, their capacity to replicate and express ribosomal subunit proteins and other cellular proteins is reduced. Furthermore, the majority of enzymes necessary for the synthesis of ATP are inactivated [64].

The reaction-time experiment proved that the UV–vis response related to the AgNP continues to change after 120 min, suggesting that the reduction and/or growth processes may not be fully equilibrated at this point. This is an important observation because if the conversion of Ag^+^ is incomplete, there will be residual dissolved silver species in the final colloid. AgNO_3_ itself showed measurable inhibition zones in the antimicrobial assay, indicating that dissolved silver precursor can contribute to antibacterial activity under the conditions used. Thus, the antimicrobial activities observed for the AgNP and Ag/MWCNT formulations cannot be ascribed to nanoparticle-specific mechanisms alone unless the residual dissolved silver content is determined independently.

The present study did not quantify residual Ag^+^ or AgNO_3_ after nanoparticle synthesis, thus cannot rule out a contribution from unreacted silver precursor to the observed antimicrobial activity. However, the larger inhibition zones for Ag/f-MWCNTs compared to AgNO_3_ suggest the antimicrobial activity of the composite formulation under the tested conditions. The relative contributions of the immobilized AgNPs, released Ag+ ions, residual precursor, and MWCNT support need to be explored further. Future work should therefore include separation/purification of the nanoparticles and quantitative analysis of dissolved silver, for example, ICP-OES, ICP-MS, or an equivalent validated analytical method, together with antimicrobial testing of purified and unpurified preparations.

The study primarily involved qualitative testing rather than a quantitative assessment of antibacterial activity, which constitutes a limitation in this research. The dimensions of inhibition zones are significantly affected by the diffusion characteristics of the agent within the agar medium, which are influenced by several factors including polarity, molecular weight, agar depth, solubility, and the conditions of incubation. [65] The study illustrates agar diffusion demonstrating activity; however, it lacks the quantitative precision necessary for determining the minimum inhibitory concentration (MIC) and does not include a growth curve analysis to elucidate the periodic behavior of the bacteria. Additional assessments involving MIC determination and time–kill kinetics are recommended to ascertain the precise minimum concentration required to inhibit growth, as well as to evaluate whether the substances exhibit bacteriostatic or bactericidal properties.

### 2.14. Antioxidant Activity Against DPPH and Hydrogen Peroxide Assay

Free radicals have been extensively studied for their significant roles in various biological processes, including cellular maturation, signaling system activation, and pathogen defense [46]. In contrast, when free radicals are present in high concentrations and persist for a long time, they can overwhelm the cellular antioxidant defenses [66]. This can lead to oxidative damage to biomolecules, which in turn can cause apoptotic and necrotic cell death [47]. Hydroxyl radicals, which are primary reactive oxygen species (ROS), have garnered significant attention due to their generation in biological systems. Hydroxyl radicals, being highly reactive, quickly oxidize both organic and inorganic substrates in close proximity to their production sites [49]. As evident from the results of this study, as summarized in Table 4, most of the MWCNT derivatives exhibited potent antiradical properties against both hydrogen peroxide and DPPH, except for pristine, unfunctionalized MWCNTs with low viability for eliminating reactive oxygen species. As such, ascorbic acid, a water-soluble compound, was used as a positive control owing to its strong free radical scavenging potential.

**Figure 7:**
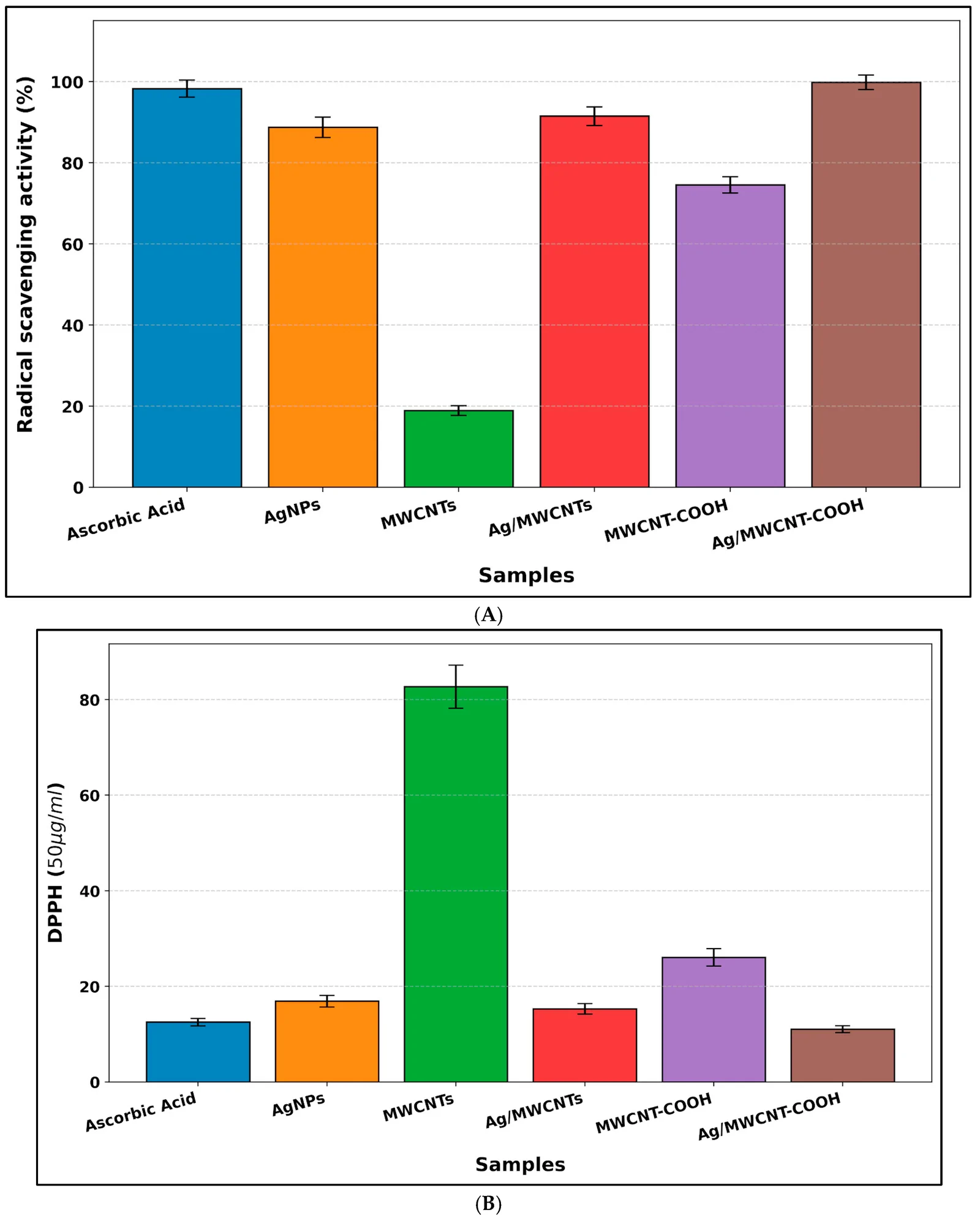

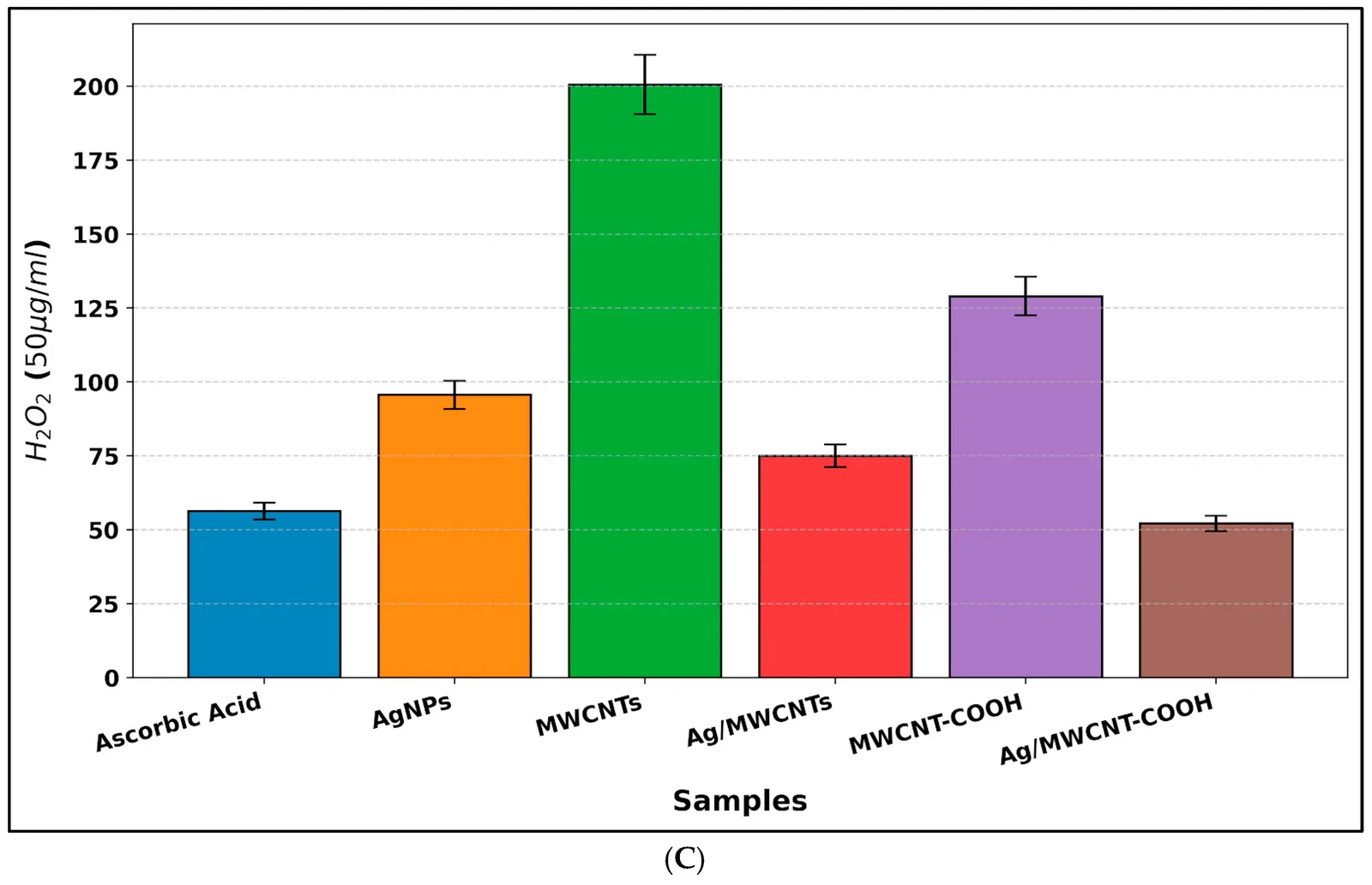
(**A**) Radical scavenging potency of the positive standard L-Ascorbic acid using the DPPH assay. (**B**) The concentration of each of the nanomaterials and nanocomposites required to scavenge 50% of the DPPH radicals. (**C**) Antioxidant activities of the prepared materials and the positive standard of L-Ascorbic Acid.

Concerning DPPH radical scavenging assays, in particular, AgNPs, functionalized MWCNTs, and their decorated nanocomposites demonstrated a potent ability to scavenge the corresponding free radicals. As regards MWCNTs, AgNP-decorated functionalized MWCNTs exhibited the strongest scavenging activity (99.82%, IC50 11.04 mg/mL), whereas pristine MWCNTs exerted the weakest (18.92%, IC50 82.89 mg/mL), as seen in Figure 7A. In terms of hydroxyl scavenging assays, AgNPs and decorated (f)MWCNT composites demonstrated considerable radical scavenging potency against the corresponding free radicals. It was also observed that ascorbic acid used as a positive control was superseded by Ag/f-MWCNTs in terms of its ability to scavenge free radicals, with IC50 values of 56.3 mg/mL and 52.15 mg/mL, respectively, as seen in Figure 7B,C. Besides the hybrid materials, MWCNT-COOH exhibited the second-lowest antioxidant activity (IC50 129.02 mg/mL), followed by the poor ability of pristine MWCNTs (IC50 200.56 mg/mL) to neutralize the free radicals.

The notable difference in antiradical properties between MWCNTs and surface-functionalized MWCNTs is attributable to crucial chemical modifications that improve the free radical scavenging activity, and the enhanced radical scavenging activity of carboxylated MWCNTs agrees with the findings of the previous study [50]. The antiradical capabilities of AgNP-decorated MWCNTs were enhanced compared to AgNPs alone. The presence of extended systems of conjugated double bonds in CNTs is responsible for their ability to donate and accept electrons. This allows MWCNTs with biosynthesized AgNPs to function as scavengers of free radicals [52]. Previous studies have demonstrated that the combination of AgNPs with MWCNTs leads to a synergistic impact, resulting in increased antioxidant activities [52,67].

To determine the estimated evaluation of the Ag content based on synthesis conditions and to compare it with analogous nanocomposites for antibacterial activity, a theoretical Ag loading was calculated from the precursor concentration utilized during the synthesis process. The theoretical maximum Ag content loading in the nanocomposite was calculated based on the synthesis parameters employed in the study, which included 2mM AgNO_3_ (250 mL) and 0.1 g of carbon nanotubes (CNTs). Upon evaluating the quantity of moles of Ag^+^ utilized, equivalent to 0.0005 moles, alongside the mass of the Ag element equivalent to approximately 53.9 mg, the theoretical maximum Ag content in the Ag/MWCNT nanocomposite is estimated to be around 35 wt%, predicated on the assumption of complete reduction and immobilization of Ag^+^. Consequently, in biological assays where identical total masses of nanomaterials were utilized, the actual quantity of Ag present in the Ag/MWCNT samples was significantly reduced compared to the AgNP-only samples. Notwithstanding the reduced Ag content, the nanocomposite demonstrated augmented antibacterial activity, indicating potential improvements in silver utilization, enhanced dispersion, or a possible synergistic interaction between AgNPs and MWCNTs, rather than merely a mass-dependent effect.

## 3. Experimental

### 3.1. Materials

The MWCNTs were purchased from Nanografi Nano Teknoloji AŞ (Ankara, Turkey) along with sulfuric acid (H_2_SO_4_, purity grade 95%) and nitric acid (HNO_3,_ purity grade 90–95%). From Sigma-Aldrich (St. Louis, MO, USA), benzoic acid, benzyl alcohol, and benzaldehyde were purchased. For pH adjustments, a solution of sodium hydroxide (NaOH) was also prepared.

### 3.2. Collection of O. dubium Bioss Plant Material

The plant material (*O. dubium*) was collected from the eastern region of the mountains, Karşıyaka (Vasileia)- Kyrenia region (35°20′40″ N and 33°7′22″ E) in Northern Cyprus during bloom in August 2022. The plant material was identified and approved by Assoc. Prof. Dr. Emmanuel Mshelia Halilu of the Department of Pharmacognosy, Faculty of Pharmacy, Cyprus International University, Cyprus. A voucher is kept with the Herbarium number CIU/PHARM/LAMI/015 in the Cyprus International University Public Herbarium. The collected leaves were washed thoroughly, dried under sunlight at an ambient temperature ranging from 20 °C to 30 °C, and crushed using a mortar and pestle to obtain the powder, which was then stored in a dry jar on a benchtop at room temperature until use.

### 3.3. Preparation of O. dubium Plant Methanolic Extract

Methanolic extraction of *O. dubium* was performed as follows. Briefly, 2.5g of the powdered biomass of the plant material was added to 25 mL of methanol (>99% purity; Sigma-Aldrich, Steinem, Germany) in a 50 mL Erlenmeyer flask and shaken at 300 rpm for 1 h 20 min at room temperature (~25 °C) on an orbital shaker. The mixture was then subjected to sonication in an ultrasound water bath at 40 °C for 30 min. The plant residue was removed by gravity filtration using a filter paper (2W, 90 mm), and the filtrate was then evaporated to dryness under vacuum at 35 °C in a rotary evaporator (Heidolph HEIVAP HL, Schwabach, Germany). The resulting residue was stored in a freezer until further tests [68].

### 3.4. Gas Chromatography-Mass Spectrometry (GC–MS) Analysis

*O. dubium* methanolic extract was analyzed using gas chromatography–mass spectrometry (GC–MS) following a method adapted from Ogbonna et al. [69]. A 30 m × 0.25 mm (thickness of film) and 0.25 μm HP-5 fused silica capillary column was utilized. Helium (99.999% purity) with a flow rate of 0.9 mL/min was used as the carrier gas. The oven temperature of the column was programmed from 50 °C (hold 1 min) to 240 °C (hold 10 min) at a 5 °C/min rate. FVME was introduced into an Agilent 7890A GC system joined with an MS (Agilent Technologies, Santa Clara, CA, USA) by auto-injection. In total, 70 eV was used to ionize the sample parts. The determination of the identities of each component was done by comparing mass spectra with records present in the National Institute of Standards and Technology (Gaithersburg, MD, USA) and Wiley (Hoboken, NJ, USA) Mass Spectrometry (MS) libraries.

### 3.5. Synthesis of Plant Extract-Mediated AgNPs

#### 3.5.1. Effect of Temperature on Synthesis of AgNPs

To explore and optimize the temperature of the reaction mixture, *O. dubium* methanolic extract was selected. The effect of temperature was studied at 40 °C, 60 °C, 80 °C, and 100 °C. 2 mL of the sample was added to 20 mL of 2 mM silver nitrate solution in a 50 mL Erlenmeyer flask. The mixture was placed on a benchtop stirrer and heated at the given parameters: 40 °C, 60 °C, 80 °C, and 100 °C. The solutions were continuously mixed using a magnetic stirrer for 30 min. The mixtures were allowed to cool down to room temperature and placed under sunlight. After 20 min, aliquots were taken to be analyzed on a UV–vis spectrophotometer as described by Abu-Hussien et al. [26]. The pH was maintained at 8.0 using NaOH solution with a pH of 12.5. The optimum temperature was selected afterwards.

#### 3.5.2. Effect of Concentration of Silver Nitrate on the Synthesis of AgNPs

A 50 mL Erlenmeyer flask was filled with 2 mL of the sample and then 20 mL of a 2 mM silver nitrate solution. The extract’s pH was consistently kept at 8.0, while the pH of the reaction mixture was continually measured. The solution was subjected to heating on a benchtop stirrer at a temperature of 40 °C for a duration of 30 min. Subsequently, it was exposed to sunlight for an additional 20 min before being analyzed for the presence of AgNPs using a UV–vis spectrophotometer. [26] The synthesis technique was repeated with several amounts of silver nitrate, namely 1 mM, 3 mM, and 5 mM. Subsequently, the optimal concentration was chosen.

#### 3.5.3. Effect of pH on the Synthesis of AgNPs

To prepare an extract at an initial pH of 8.0, NaOH was added dropwise using a pipette into 5 mL of plant methanolic extract, monitored with a pH meter. 2 mL of the plant extract was added to 20 mL of 2 mM silver nitrate solution in a 50 mL Erlenmeyer flask and vigorously mixed at 40 °C for four minutes on a workbench. The combination was cooled using sunlight for a duration of 20 min, and a UV measurement was made after exposure to UV radiation. This procedure was repeated for the plant extract at pH 9, 10, and 11, and the optimum pH was determined [27].

#### 3.5.4. Effect of Volume of Plant Extract on the Synthesis of AgNPs

The experiment involved adding 2 mL of the plant extract to a 20 mL solution of 2mM silver nitrate. The mixture was then heated at 40 °C for 30 min while being continuously stirred. The solution’s pH was adjusted to 8.0, and the formation of AgNPs was observed through the color change from pale yellow to dark orange. The nanoparticles synthesized using green methods were exposed to UV irradiation for 20 min in sunlight, and the UV–vis spectrum was subsequently recorded. The experiment was replicated using plant extract volumes of 4 mL, 6 mL, and 8 mL [26]. Subsequently, the optimal volume of plant extract was chosen.

#### 3.5.5. Effect of Reaction Time on the Synthesis of AgNPs

A 50 mL Erlenmeyer flask containing 2 mL of plant methanolic extract and 20 mL of 2 mM silver nitrate was used to create the AgNPs. The flask was filled with a magnetic stir bar, placed on a tabletop stirrer, and heated for 30 min at 40 °C. The same process was run for 60, 90, and 120 min in order to examine the impact of various time intervals on the reduction of silver ions. The reaction was allowed to reach room temperature and was then exposed to sunlight for a duration of 20 min [26]. After the thermal reaction stage, the reaction mixtures were exposed to natural sunlight for the irradiation time defined before UV–vis analysis. In the present work, sunlight was used as the irradiation source instead of a calibrated monochromatic UV lamp, so that wavelength distribution and irradiance were not controlled independently. The experiment of irradiation time was thus designed to assess the operational effect of the exposure time under the same environmental irradiation conditions rather than to establish a wavelength-specific photochemical mechanism. The UV–vis spectrophotometer was used to examine the production of NPs. A certain reaction period that maximizes the production of nanoparticles was chosen.

### 3.6. Functionalization of MWCNTs

**Figure 8:**
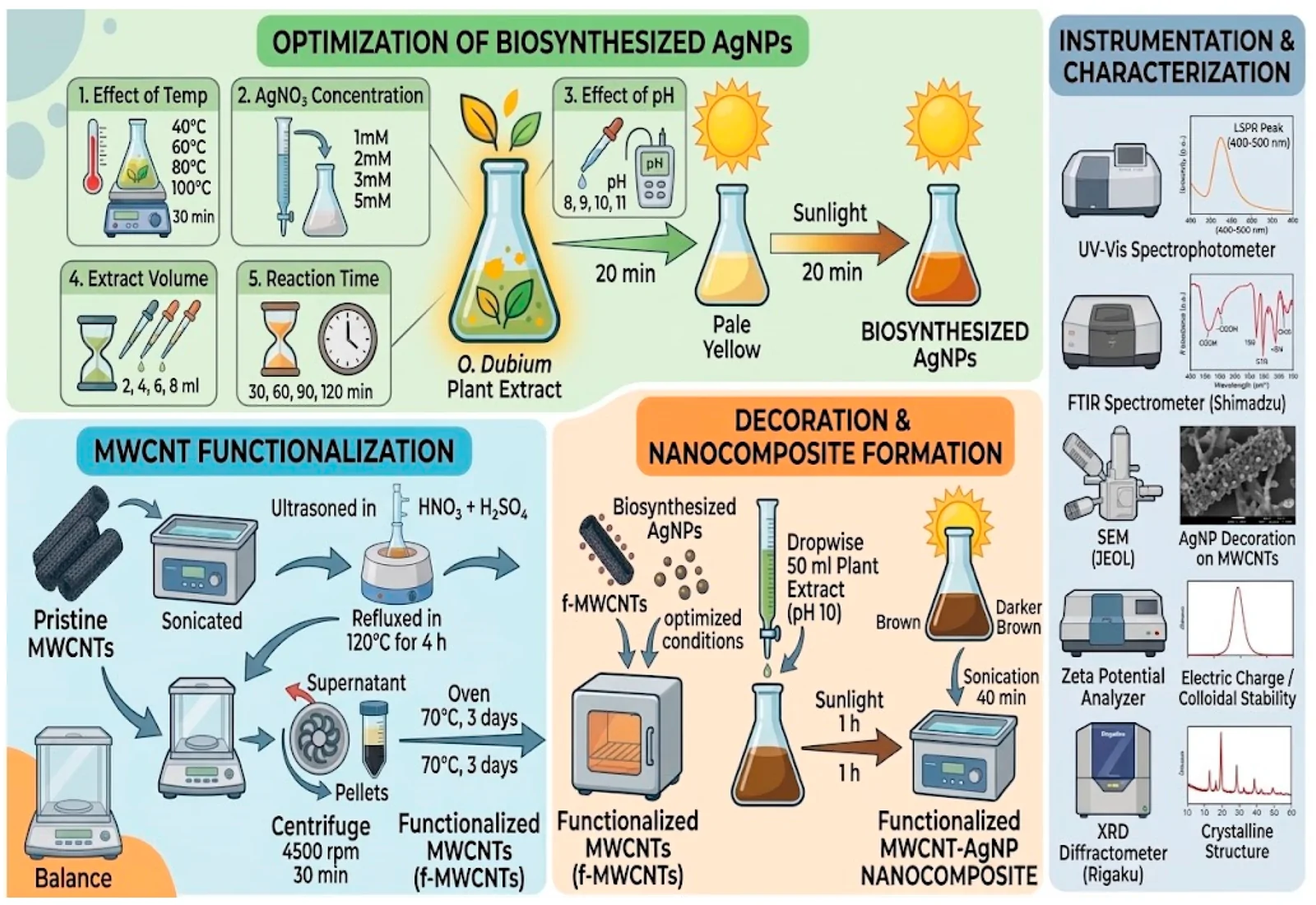
Schematic illustration of the preparation of functionalized MWCNTs and their biosynthesized AgNP–decorated nanocomposite structures.

An acid-assisted chemical oxidation method was employed to introduce oxygen-containing functional groups onto the surface of pristine MWCNTs. Briefly, 0.5 g of pristine MWCNTs was accurately weighed and refluxed under continuous stirring in an H_2_SO_4_/HNO_3_ acid mixture (3:1, *v*/*v*; H_2_SO_4_, 95–97%; HNO_3_, 65%) at 120 °C for 4 h to prepare OH- and/or COOH-functionalized MWCNTs. Prior to refluxing, the MWCNT suspension was sonicated to ensure uniform dispersion of the nanotubes in the colloidal medium, as shown in Figure 8. Subsequently, the reaction mixture was diluted with 1 L of distilled water and allowed to stand for 3 days. The resulting suspension was then centrifuged at 4500 rpm for 30 min to collect the functionalized MWCNTs as pellets. The supernatant was discarded, and the resulting pellets were collected and dried in an oven at 70 °C [28].

### 3.7. Decoration of Multi-Walled Carbon Nanotube Hybrids

A plant extract-mediated green synthesis method was employed for the in situ formation and decoration of AgNPs onto pristine MWCNTs and functionalized MWCNTs (f-MWCNTs). The method was based on the optimized conditions previously established for the synthesis of AgNPs using *Origanum dubium* plant extract as a natural reducing and stabilizing agent, instead of conventional chemical reducing agents such as DMF or sodium borohydride [28]. Briefly, 0.1 g of CNTs was dispersed in 250 mL of 2 mM silver nitrate (AgNO_3_) solution, which served as the silver precursor. Subsequently, 50 mL of plant extract adjusted to pH 10 was added dropwise using a burette. A color change from brown to dark brown was observed, indicating the reduction of silver ions and formation of AgNPs. The reaction was allowed to proceed for 1 h and was subsequently exposed to sunlight for an additional 1 h to facilitate AgNP formation. The resulting AgNP/CNT suspension was then sonicated for 40 min at room temperature to promote uniform dispersion of the AgNPs and their deposition onto the CNT surfaces.

### 3.8. Characterization

The AgNPs were studied using UV–vis spectroscopy (Shimadzu UV-2450, Kyoto, Japan) to verify the existence of LSPR and a blue shift in the peak into the 400–500 nm range in the visible region of the spectrum. The functional groups of the samples were detected using FTIR (Shimadzu IR-Prestige 21). The surface morphology of the MWCNTs was determined using SEM (JEOL JSM-6610LV, Tokyo, Japan). The magnitude of the electric charge was measured using Zeta potential (ATOMIKA TEKNIK, Ankara, Turkey). The crystalline or amorphous nature of the samples was determined by XRD (Rigaku Ultima IV, Tokyo, Japan).

### 3.9. Antimicrobial Activity

The antibacterial activity of the synthesized AgNPs and their CNT-based nanocomposites was assessed against two representative bacterial strains with distinct cell wall structures: methicillin-resistant *Staphylococcus aureus* (MRSA), representing Gram-positive bacteria, and *Escherichia coli* (*E. coli*), representing Gram-negative bacteria, using the well-diffusion method [29]. The biosynthesized AgNPs and their MWCNT-based nanocomposites were evaluated for their antibacterial activities against two bacterial reference strains, methicillin-resistant *Staphylococcus aureus* (MRSA; ATCC 43300, strain F-182) as a Gram-positive bacterium and *Escherichia coli* (ATCC 25922, FDA strain Seattle 1946) as a Gram-negative bacterium. *S. aureus* ATCC 43300 is a clinical MRSA isolate originally obtained from Kansas, USA, and is characterized as SCCmec type II and resistant to methicillin and oxacillin. *E. coli* ATCC 25922 is a clinical isolate originally obtained from Seattle, WA, USA, and is a standard reference strain commonly used for antimicrobial susceptibility testing. All bacterial conditions were assayed in triplicate. A Mueller–Hinton agar was prepared as per the manufacturer’s instructions and sterilized at 121 °C for 30 min. The surface of the agar was inoculated with a bacterial suspension of about 107 CFU/mL. Aseptically, wells of 0.8 cm diameter were made using a sterile cork borer. Then, 105 μL of each test material at a concentration of 200 μg/mL was added to the respective wells. Thus, each well received 21 μg of total test material. The materials tested were methanolic extract of *O. dubium* plant, AgNPs, pristine MWCNTs, acid-functionalized MWCNTs (f-MWCNTs), Ag/MWCNTs, and Ag/f-MWCNTs. Antibiotic disks were used as a positive control and sterile distilled water as a negative control. Plates were incubated at 37 °C for 24 h, and the diameter of each inhibition zone was measured. The diameter of the inhibition zone was calculated by subtracting the diameter of the agar well from the total diameter of the inhibition zone.

### 3.10. Antioxidant Activity Against DPPH

Antioxidant activity of *O. dubium* was determined by the DPPH test [30]. The methanolic plant extracts were prepared in different concentrations (0.002–20 mg/mL). The stock solution of 0.1 mM DPPH was prepared using 3.94 mg of powdered DPPH in 100 mL of pure ethanol. The ethanol solution of DPPH (2 mL) was added to 2 mL of the plant extract at different concentrations, as well as their respective solvents, in the dark by covering the Erlenmeyer flask with aluminum foil. The reaction was conducted at a temperature of 50 °C using a stirrer placed on a tabletop. The absorbance of the reaction was measured at a wavelength of 517 nm (As). The absorbance at a wavelength of 517 nm was measured for a pure ethanol solution of the DPPH radical, which was synthesized as described above (Ac). Prior to treatment with DPPH, the plant extracts and their respective AgNP solutions were tested for the absorbance values at 517 nm (AB). Free radical scavenging activity was calculated using Equation (1).

(1) $$DPPH\;Scavenging\;activity=\frac{AB-AS}{AB}\times 100\%$$

where AB and AS are the absorbances of the blank and sample, respectively.

### 3.11. Antioxidant Activity with Hydrogen Peroxide Assay

A 2 mL volume of aqueous leaf extract and optimized concentrations (50, 100, 200, and 400 µg/mL) of AgNPs were separately added to Erlenmeyer flasks containing 2 mL of a 100 mM H_2_O_2_ solution prepared in phosphate buffer with a pH of 7.4. The mixtures were shaken and incubated in the dark for 20 min on a hot plate set to 40 °C. The flasks were shielded with aluminum foil to prevent the ingress of photons into the reaction mixture, thereby suppressing any concurrent side reactions. L-ascorbic acid (1 mg/mL) was prepared in ethanol in different concentrations to be used as a standard for a reliable comparison across the variety of concentrations [28,30]. Absorbance was taken at 230 nm using a UV–vis spectrophotometer, and percentage inhibition was calculated using a formula similar to the DPPH scavenging activity formula (Equation (2)) [30].

(2) $$\%\;Inhibition=\frac{AB-AS}{AB}\times 100\%$$

where AB and AS are the absorbances of the blank and sample, respectively.

## 4. Conclusions

In this study, AgNPs were synthesized using *Origanum dubium* plant extract as a biological reducing and capping system and then deposited on pristine and acid-functionalized MWCNTs. The synthesis conditions were optimized for the reaction temperature, AgNO_3_ concentration, plant extract concentration, pH, reaction time, and irradiation time. The obtained materials were characterized by UV–vis spectroscopy, FTIR, SEM, DLS/zeta-potential analysis, and XRD. The characterization data collectively confirmed the formation of AgNPs and their deposition over MWCNT-based supports. The functionalized MWCNT system showed better colloidal properties than the pristine system. The MWCNT composites containing AgNPs showed larger inhibition zones against the bacterial strains tested than some of the individual components, and the Ag/f-MWCNT formulation showed the highest values of inhibition zones in the conditions investigated. The materials also showed radical-scavenging activity in the DPPH and hydrogen peroxide assays. However, the agar-diffusion experiments are evidence of antimicrobial activity and not a quantitative determination of minimum inhibitory concentration or bactericidal kinetics. Furthermore, measurable inhibition zones were produced in the presence of AgNO_3_ itself, and residual dissolved silver was not directly quantified after synthesis; therefore, the contribution of residual Ag+ or AgNO_3_ to the antimicrobial response cannot be completely excluded. These findings indicate that combining biosynthesized AgNPs with functionalized MWCNTs can enhance the antimicrobial and antioxidant performance of the resulting hybrid system. Although the present study demonstrated enhanced biological activities, the molecular mechanisms responsible for these effects were not directly investigated. In particular, Ag^+^ release kinetics, nanoparticle–cell interactions, intracellular oxidative stress pathways, and potential cytotoxic effects were beyond the scope of the present work. Therefore, future studies should investigate these mechanisms and evaluate the biological safety of the Ag/f-MWCNT nanocomposites in relevant mammalian cell models.

The present study demonstrates the feasibility of *O. dubium* extract for the plant-mediated synthesis of AgNP/MWCNT nanocomposites. However, complete mechanistic insight into the synthesis has to be further explored. Therefore, future work should couple further spectroscopic analysis with quantitative elemental and chemical characterization (including techniques able to resolve the oxidation state and coordination environment of silver) and theoretical calculations to clarify the interactions between phytochemical constituents, Ag+, AgNP surfaces, and functionalized MWCNTs. Quantification of residual dissolved silver, SEM, nanoparticle purification studies, MIC determination, and time–kill assays would also help to distinguish nanoparticle-associated effects from those arising from dissolved silver species. These studies would provide a more robust mechanistic and quantitative basis for assessing the potential applications and safety of these nanocomposites.

## Figures and Tables

**Table 1:** Gas chromatography-mass spectrophotometry (GC-MS) examination of the methanolic leaf extract of *O. dubium* revealed the most prevalent phytochemical bioactive compounds.

Compound (IUPAC-Name)	Retention Time (Min.)	Peak Area%	Molecular Formula	Nature of Compound
p-Cymene-2,5-diol	11.213	0.19	C_10_H_14_O_2_	*Terpenes*
Thymoquinone	7.464	0.25	C_10_H_12_O_2_	*Quinones*
Lidocaine	15.028	0.23	C_14_H_22_N_2_O	*Amides*
Phenol, 2,2’-methylenebis [6-(1,1-dimethylethyl)-4-methyl-	19.806	0.42	C_23_H_32_O_2_	*Phenolics*
3-Methyl-4-isopropylphenol	8.045	3,98	C_10_H_14_O	*Phenolics*
2,4-Di-tert-butyl phenol	10.691	0.10	C_14_H_22_O	*Phenolics*
4-Cyanophenol	20.634	0.95	C_7_H_5_NO	*Phenolics*
Benzyl alcohol	4.791	0.11	C_6_H_5_CH_2_OH	*Alcohols*

**Table 2:** DLS analysis summarizing the size distribution by intensity of synthesized AgNPs, pure and modified MWCNTs, and biosynthesized AgNPs deposited on the walls.

The Sample	Zeta Potential (mV)	Zeta-Size (nm)	Polydispersity Index (PI)	Colloidal State
Methanol AgNPs	−22.58	33.52	0.6144	Moderately stable (minimal aggregation)
Pristine MWCNTs	−12.18	230.9	0.6307	Unstable (reasonable aggregation)
Ag/MWCNT	−21.3	97.16	0.6704	Moderately stable (minimal aggregation)
MWCNT-COOH	−17.95	135.5	0.1419	Fairly Stable (potential aggregation)
Ag/MWCNT-COOH	−38.93	73.61	0.3626	Highly stable (negligible agglomeration)

**Table 3:** Inhibitory activities of biosynthesized AgNPs against Gram-positive MRSA and Gram-negative *E. coli* ± S.D of three replicates.

	Inhibition Zones (cm)	
	*S. aureus* MRSA	*E. coli*
Nanomaterials	MRSA	*E. coli*
Plant Extract	0.311 ± 0.011	0.375 ± 0.03
AgNPs	0.566 ± 0.027	0.509 ± 0.029
MWCNTs	0.226 ± 0.052	0.127 ± 0.023
f-MWCNTs	0.916 ± 0.047	1.09 ± 0.014
Ag/MWCNTs	1.281 ± 0.069	1.302 ± 0.13
Ag/f-MWCNTs	1.806 ± 0.021	1.661 ± 0.076
AgNO_3_	0.562 ± 0.062	0.73 ± 0.019
Negative Control	0 ± 0	0 ± 0
Cefoxitin	1.12 ± 0.03	1.071 ± 0.034
Ampicillin	0.362 ± 0.026	0.289 ± 0.041

**Table 4:** The summary of minimum inhibitory concentrations of the standard and the biosynthesized nanomaterials required to neutralize 50% of the DPPH (2,2-diphenyl-1-picrylhydrazyl) and hydrogen peroxide radicals.

Free Radical Scavenging Assays	DPPH IC50 (µg/mL)	H2O2 IC50 (µg/mL)
Ascorbic Acid	12.50	56.30
AgNPs	16.89	95.62
MWCNTs	82.69	200.56
Ag/MWCNTs	15.27	75.02
MWCNT-COOH	26.07	129.02
Ag/MWCNT-COOH	11.04	52.15

## Data Availability

The raw data supporting the conclusions of this article will be made available by the authors on request.
